# Synthesis and characterisation of new antimalarial fluorinated triazolopyrazine compounds

**DOI:** 10.3762/bjoc.19.11

**Published:** 2023-01-31

**Authors:** Kah Yean Lum, Jonathan M White, Daniel J G Johnson, Vicky M Avery, Rohan A Davis

**Affiliations:** 1 Griffith Institute for Drug Discovery, School of Environment and Science, Griffith University, Nathan, QLD 4111, Australia; 2 School of Chemistry and Bio21 Institute, The University of Melbourne, Melbourne, VIC 3010, Australia; 3 Discovery Biology, Griffith University, Nathan, QLD 4111, Australia; 4 NatureBank, Griffith University, Nathan, QLD 4111, Australia

**Keywords:** antimalarial, characterisation, Diversinate^TM^, fluorine, triazolopyrazine, scaffold, Open Source Malaria

## Abstract

Nine new fluorinated analogues were synthesised by late-stage functionalisation using Diversinate™ chemistry on the Open Source Malaria (OSM) triazolopyrazine scaffold (Series 4). The structures of all analogues were fully characterised by NMR, UV and MS data analysis; three triazolopyrazines were confirmed by X-ray crystal structure analysis. The inhibitory activity of all compounds against the growth of the malaria parasite *Plasmodium falciparum* (3D7 and Dd2 strains) and the cytotoxicity against a human embryonic kidney (HEK293) cell line were tested. Some of the compounds demonstrated moderate antimalarial activity with IC_50_ values ranging from 0.2 to >80 µM; none of the compounds displayed any cytotoxicity against HEK293 cells at 80 µM. Antimalarial activity was significantly reduced when C-8 of the triazolopyrazine scaffold was substituted with CF_3_ and CF_2_H moieties, whereas incorporation of a CF_2_Me group at the same position completely abolished antiplasmodial effects.

## Introduction

Malaria is an infectious disease caused by *Plasmodium* parasites and is a major global threat to human health. The WHO World Malaria Report 2021, estimates 241 million cases of malaria and 627,000 deaths globally in 2020, an increase of 12% from the previous year [1]. The increase was mainly from countries in the WHO African region, which accounted for about 95% of malaria cases and deaths, and was associated with service disruptions during the COVID-19 pandemic [1]. Infants and young children are at a disproportionately high risk of severe malaria and death, as 80% of deaths in this region were children under five [1]. Whilst there are drugs available for the treatment of malaria infections, most have now succumbed to parasite drug resistance and thus reduced clinical efficacy [2–3]. Consequently, new antiplasmodial drugs with novel malaria targets are urgently needed to combat the global problem of parasite drug resistance. For more than 10 years, the Open Source Malaria (OSM) consortium [4] has had an interest in identifying and developing novel antimalarial compounds that belong to a variety of chemotypes, one of which includes the 1,2,4-triazolo[4,3-*a*]pyrazine scaffold [5]. This particular series, known as OSM Series 4, has demonstrated significant potency against various strains of *Plasmodium falciparum* (*Pf*) with IC_50_ values as low as 16 nM. The series also showed decent in vitro human liver microsome and human hepatocyte stability, with hepatic intrinsic clearance of <8.1 µL/min/mg [5]. Furthermore, minimal poly-pharmacology and cytotoxicity have been identified for this series to date, giving confidence in its specificity and tolerability, and thus supporting on-going efforts towards the continued development of this unique antimalarial structure class [5]. Through investigations into the mechanism of action of OSM Series 4 compounds, it has been suggested that this nitrogen-rich chemotype inhibits the ATPase, *Pf*ATP4 [6]. *Pf*ATP4 functions as a Na^+^/H^+^-ATPase, which allows the malaria parasite to regulate Na^+^ to maintain cell homeostasis [7–9]. Interfering with this process means the parasite is unable to regulate Na^+^ [10], resulting in a significant increase in the acid load of the cell, which can lead to parasite growth inhibition and ultimately parasite death [8–9]. One of the current Series 4 aims includes lead optimisation to improve solubility and metabolic stability while retaining potency [5]. Late-stage functionalisation (LSF) is a strategy involving the use of C–H bonds as chemical handles for the introduction of various functional groups, which has been widely employed by medicinal chemists to generate new analogues of lead compounds without the need for de novo synthesis [5]. Baran et al. has developed an operationally simple, radical-based functionalisation strategy that allows direct transformation of C–H bonds to C–C bonds in a practical manner [11]. This strategy involves the utilisation of sodium and zinc sulfinate-based reagents (marketed by Merck as Diversinates™) to functionalise heteroaromatic C–H bonds of unprotected systems in a variety of solvents at room temperature and without the requirement of an inert atmosphere or solvent purification [11–13].

In our previous work on OSM Series 4 scaffolds [14], we had undertaken some preliminary investigations into the use of commercially available Diversinate™ reagents and showed the bicyclic nitrogen-rich core of Series 4 was amenable to this chemistry, with radical sulfinate substitution occurring with high-selectivity at C-8 and in respectable yields. This paper reports additional and more thorough Diversinate™ studies on three phenethyl ether substituted triazolopyrazine scaffolds, with a particular focus on incorporating the fluoro fragments -CF_3_, -CF_2_H and -CF_2_Me into the OSM Series 4 structures via LSF chemistry. The new library of triazolopyrazines were all evaluated in vitro for antimalarial activity and cytotoxicity.

## Results and Discussion

Previous structure–activity relationship (SAR) studies reported that any substitution at the C8 position of Series 4 triazolopyrazines can lower the potency for *P. falciparum* [14–16]. However, a recent preliminary SAR study identified that substitution at the C8 position with trifluoromethane and difluoroethane moieties using Diversinate™ chemistry increased the potency of the parent scaffold (compound **2**), suggesting the potential of these fluoroalkyl groups for improving the potency of other promising leads within the OSM project [14]. Fluorine-containing compounds have exhibited wide applications in pharmaceuticals and agrochemicals – approximately 20% of marketed drugs are fluoro-pharmaceuticals, while for agrochemicals, 53% are fluoro-compounds [17–18]. In recent decades, the introduction of fluorine or a fluorinated functional group into organic compounds has become increasingly prevalent in drug design and development, as fluorine substitution can greatly influence drug potency, pharmacokinetic and pharmacodynamic properties [19]. Therefore, in this study we undertook additional LSF investigations by introduction of fluoroalkyl groups to OSM leads with the aim to probe the SAR of 8-fluoroalkylated triazolopyrazine derivatives and further improve their potency.

**Scheme 1:**
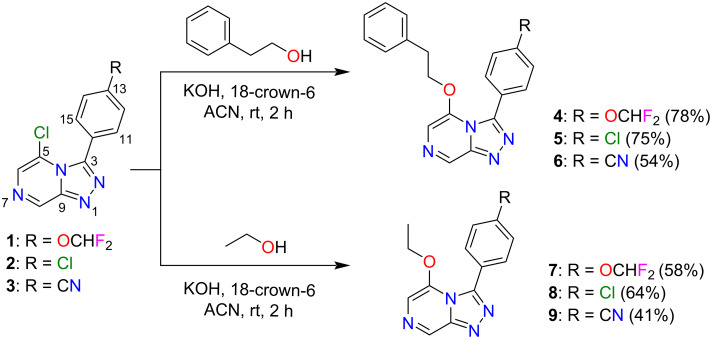
Synthesis of ether triazolopyrazine derivatives **4**–**9**.

Based on the existing SAR data for the C3 position of the triazolopyrazine core, substituents with a phenyl ring containing alkyl, cyano, nitro, or halogenated groups at the *para*-position were crucial for activity [5,16,20]. Thus, compounds **1**–**3** with a *para-*phenyl-OCHF_2_, -Cl or -CN substituent at the C3 position of the triazole were selected as scaffolds in this study. In addition, the reported SAR data also indicated that the use of an ether linker on the pyrazine ring, with a two methylene unit chain length between the heterocyclic core and benzylic substituent, improved the potency of these compounds [16]. Hence, scaffolds **1**–**3** were then converted into a series of ether-linked triazolopyrazines with phenethyl alcohol or ethanol using the standard nucleophilic displacement method as previously described (Scheme 1) [14,16]. Structures of synthesised compounds **4**–**9** were determined using 1D/2D NMR and HRMS (Supporting Information File 1, S6–S23). Crystals of compounds **5** and **6** were also analysed by X-ray crystallography studies (Supporting Information File 1, S51, S52, and S54), which confirmed the structure assignment. Compounds **4**–**6** were known OSM compounds that displayed good selective activity with IC_50_ values of <1 µM [16,21], whereas **7**–**9** are new ether derivatives without a phenyl ring that were synthesised for SAR evaluation.

**Scheme 2:**
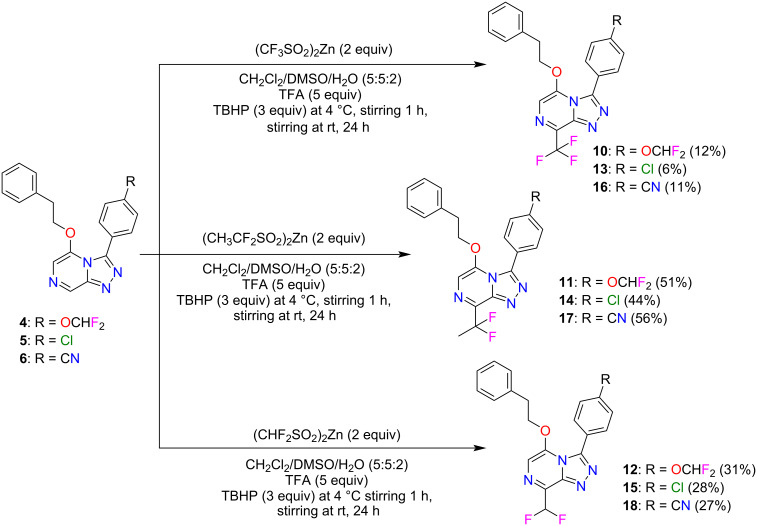
Synthesis of fluorinated triazolopyrazine compounds (**10**–**18**) via Diversinate™ chemistry.

The incorporation of fluoroalkyl groups at the C8 position of three OSM leads (**4**–**6**) was performed using Diversinate™ chemistry following the previously described method (Scheme 2) [14]. The Diversinate™ reagents used in this study were zinc trifluoromethanesulfinate (TFMS), sodium 1,1-difluoroethanesulfinate (DFES) and zinc difluoromethanesulfinate (DFMS). In brief, a mixture of the respective scaffold, Diversinate™ (2 equiv), and TFA (5 equiv) in DMSO/CH_2_Cl_2_/H_2_O (5:5:2) was stirred for 30 min at room temperature and cooled to 4 °C. Then, aqueous *tert*-butyl hydroperoxide (TBHP, 70%, 3 equiv) was slowly added over 5 min and stirring continued for 1 h. The mixture was slowly warmed to room temperature with stirring for another 24 h. The products were isolated and purified using C_18_ and phenyl HPLC (MeOH/H_2_O/0.1% TFA).

**Figure 1:**
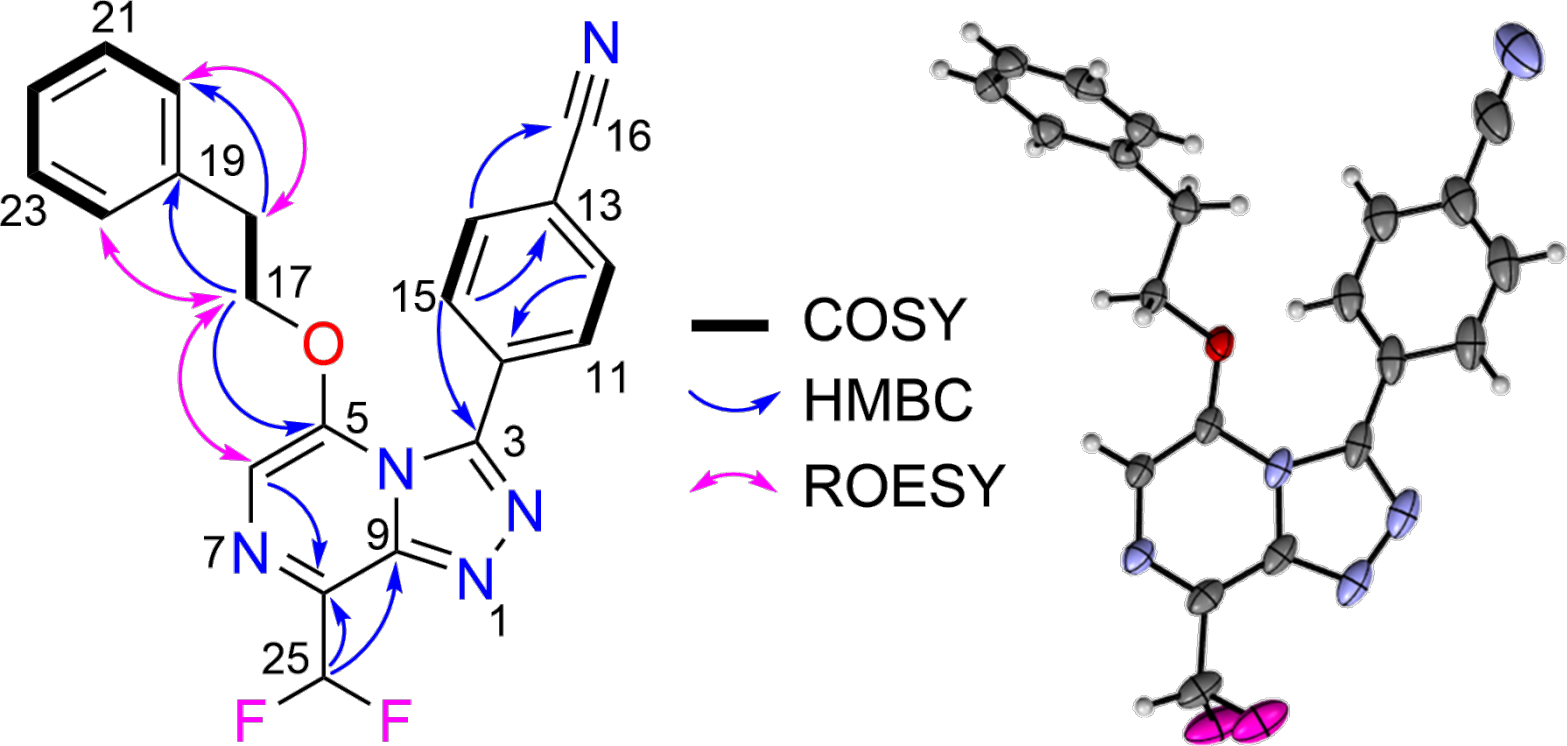
Key HMBC, COSY and ROESY correlations, and ORTEP drawing of **18**.

The structures of all fluorinated triazolopyrazine compounds (**10**–**18**) were fully characterised using 1D/2D NMR and HRMS (Supporting Information File 1, S24–S50). The structure elucidation studies on a fluorinated triazolopyrazine are detailed below. The ^1^H NMR spectrum of **18** in CDCl_3_ (Supporting Information File 1, S49) revealed signals corresponding to two methylenes [δ_H_ 2.99 (H-18) and 4.60 (H-17)] and 10 aromatic protons [δ_H_ 7.49 (H-6), 7.68 (H-11, H-15), 7.64 (H-12, H-14), 6.90 (H-20, H-24), 7.26 (H-21, H-22, H-23)]. A triplet resonating at δ_H_ 7.06 represents the proton located in the difluoromethyl group (H-25), with a ^1^*J*_HF_ coupling constant of 53.4 Hz. The ^13^C NMR spectrum revealed a ^2^*J*_CF_ triplet splitting (27.3 Hz) for δ_C_ 137.5, which allowed the assignment of C-8. The substitution of a difluoromethyl moiety at the C8 position of the triazolopyrazine ring was further supported by HMBC correlations of H-25 to δ_C_ 137.5 (C-8) and δ_C_ 144.6 (C-9). Detailed analysis of the HMBC spectrum also confirmed the presence of the phenyl ether sidechain at C-5 of the pyrazine ring, based on correlations from the aromatic protons δ_H_ 6.90 (H-20, H-24) to δ_C_ 34.3 (C-18), and δ_H_ 4.60 (H-17) to δ_C_ 135.5 (C-19) and 145.0 (C-5). This assignment was further confirmed by ROE correlations from H-17 to H-20, H-24 and H-6 (δ_H_ 7.49). The presence of a benzonitrile ring at the C-3 position was supported by the HMBC correlations of aromatic protons δ_H_ 7.68 (H-11, H-15) to C-3 (δ_C_ 146.2), and δ_H_ 7.64 (H-12, H-14) to C-16 (δ_C_ 118.0). Furthermore, crystals obtained for compound **18** were analysed by X-ray crystallographic studies and confirmed the NMR-based structure assignment. Key COSY, HMBC, and ROESY correlations, and ORTEP drawing of compound **18** are shown in Figure 1.

All compounds were tested for their antimalarial activity against *P. falciparum* 3D7 (chloroquine-sensitive strain) and Dd2 (chloroquine, pyrimethamine and mefloquine drug-resistant strain) (Table 1). In terms of the cLog*P* values of these compounds, an increase in hydrophobicity did not improve the potency. A similar trend was also observed in a previous study [20], where an increase in the hydrophobicity of several triazolopyrazine derivatives resulted in significant drops in antimalarial activity. However, the same paper also commented that the cLog*P* values showed no significant correlation with experimental potency when compared to other Series 4 triazolopyrazines [20]. In addition, consistent with reported SAR data [14,16,21], the ether-linked compounds **4**–**6** exhibited strong activity with IC_50_ values of 0.2–1.2 µM, whereas compounds **7**–**9** with the removal of the phenyl ring from the ether methylene group resulted in a loss of potency at the tested concentrations. For fluorinated compounds, previous studies [14] reported that the introduction of CF_3_ and CF_2_Me groups at the C-8 position of scaffold **2** improved the antimalarial activity. In particular, compounds with a CF_2_Me moiety showed a 7.3-fold improvement in potency (IC_50_ = 1.7 µM) compared to the parent scaffold **2** (IC_50_ = 12.6 µM) [14]. Herein, ether-linked triazolopyrazine scaffolds with CF_3_ or CF_2_H moieties at the C-8 position displayed weak antimalarial activity, whereas incorporation of a CF_2_Me group completely abolished the effect at the tested concentrations, in comparison to the parent scaffolds **4**–**6**. These data suggest that substituents at the C-5 position of the triazolopyrazine core appear to influence the antimalarial activity of C-8 fluoroalkyl-substituted compounds. Additional investigations on other OSM leads are warranted to further expand the SAR surrounding the 8-position of Series 4 triazolopyrazines with fluoroalkyl substituents or other functional groups. In addition, the replacement of H-8 by small electron-withdrawing groups appeared to be detrimental for activity in Series 4 compounds.

**Table 1:** Biological data for triazolopyrazine analogues **1**–**18**.

Compound	cLog*P*	*Pf* 3D7^a^ IC_50_ ± SD µM	*Pf* Dd2^b^ IC_50_ ± SD µM	SI for 3D7^c^	SI for Dd2^c^

**1**	1.7	45.20 ± 5.42^d^	34.48 ± 4.46^d^	>2	>2
**2**	2.2	18.91 ± 0.63^d^	17.08 ± 0.43^d^	>4	>4
**3**	1.4	35.46 ± 3.33^d^	26.16 ± 1.01^d^	>2	>3
**4**	3.2	0.24 ± 0.01	0.26 ± 0.01	>329	>312
**5**	3.6	0.20 ± 0.02	0.22 ± 0.01	>396	>364
**6**	2.8	1.15 ± 0.05	1.17 ± 0.09	>70	>68
**7**	1.7	^e^	^e^	–	–
**8**	2.1	2.72 ± 0.03	2.91 ± 0.18	>29	>27
**9**	1.4	NA	NA	–	–
**10**	4.1	11.35 ± 1.45^d^	17.08 ± 0.71^d^	>7	>4
**11**	4.1	NA	NA	–	–
**12**	3.9	16.38 ± 1.13^d^	19.55 ± 0.42^d^	>5	>4
**13**	4.5	9.43 ± 0.44	10.24 ± 0.75	>9	>8
**14**	4.6	NA	NA	–	–
**15**	4.3	18.77 ± 0.21^d^	14.29 ± 2.26^d^	>4	>6
**16**	3.7	22.53 ± 1.03^d^	45.09 ± 6.58^d^	>4	>2
**17**	3.8	^f^	^f^	–	–
**18**	3.6	31.04 ± 2.37^d^	58.56 ± 1.68^d^	>3	>1

Control		*Pf* 3D7^a^ IC_50_ ± SD nM	*Pf* Dd2^b^ IC_50_ ± SD nM	SI for 3D7^c^	SI for Dd2^c^

Pyrimethamine		15.20 ± 1.20	^f^	>657	–
Artesunate		2.90 ± 0.10	2.40 ± 0.10	>857	>1051
Puromycin		79.00 ± 5.70	101.80 ± 9.50	127	98

^a^3D7 = *P. falciparum* (chloroquine-sensitive strain); ^b^Dd2 = *P. falciparum* (chloroquine, pyrimethamine and mefloquine drug-resistant strain); ^c^All compounds **1**–**18** and controls tested for cytotoxicity against human embryonic kidney cells (HEK293) in order to determine selectivity index (SI) using the formula: SI = HEK293 IC_50_/parasite IC_50_, all compounds were inactive towards HEK293 at the top dose of 80 µM; ^d^Estimated IC_50_ as a plateau of inhibition was not reached; SD = standard deviation. All cLog*P* values were calculated using the open-source program DataWarrior [22]. ^e^<81% inhibition observed at the top dose of 80 µM; ^f^<33% inhibition observed at the top dose of 80 µM; NA = not active at the top dose of 80 µM.

## Conclusion

Three selected Series 4 triazolopyrazine scaffolds (**1**–**3**) were converted to three known (**4**–**6**) and three new ether-linked derivatives (**7**–**9**). H-8 of the known OSM leads **4**–**6** was subsequently substituted with three different fluoroalkyl moieties using Diversinate™ chemistry that resulted in the synthesis of nine new fluorinated triazolopyrazines (**10**–**19**). The antimalarial data indicated that substitution of H-8 of ether-linked triazolopyrazines with fluoroalkyl moieties led to a reduction or loss of activity at the tested concentrations. These data indicate that additional medicinal chemistry efforts involving H-8 replacement with fluoroalkyl groups for this OSM series is not warranted.

## Experimental

**General procedure for Diversinate™ derivatisations on triazolopyrazine scaffolds.** In a manner analogous to [14], the scaffold (0.1 mmol) was dissolved in DMSO/CH_2_Cl_2_ (1:1, 250 µL:250 µL) before the addition of Diversinate™ (0.2 mmol, 2 equiv), TFA (40 μL, 0.5 mmol, 5 equiv) and filtered H_2_O (100 μL). The reaction mixture was stirred for 30 min at room temperature, cooled to 4 °C then 70% TBHP solution (41 μL, 0.3 mmol, 3 equiv) was slowly added over 5 min and left to stir for 1 h at 4 °C. The mixture was then slowly warmed to room temperature with stirring for 24 h. The crude reaction mixture was dried down initially under nitrogen then reduced under vacuum before being re-dissolved in MeOH/CH_2_Cl_2_ (1:1, 500 µL:500 µL) then preadsorbed to C_18_-bonded silica (≈1 g). The resulting material was packed into a guard cartridge that was subsequently attached to a semipreparative C_18_-bonded silica HPLC column. Isocratic conditions of 30% MeOH/70% H_2_O (0.1% TFA) were held for the first 1 min, followed by a linear gradient to 100% MeOH (0.1% TFA) over 59 min, all at a flow rate of 9 mL/min. Sixty fractions (60 × 1 min) were collected from the start of the HPLC run. Fractions containing UV-active material were analysed by ^1^H NMR spectroscopy and LCMS, and relevant fractions with desired products were combined. In order to afford the desired products in sufficient purity (>95%) for biological testing, the combined fractions were further purified using semipreparative phenyl HPLC with isocratic conditions of 50% MeOH/50% H_2_O (0.1% TFA) held for the first 1 min, followed by a linear gradient to 80% MeOH/20% H_2_O (0.1% TFA) over 59 min, all at a flow rate of 9 mL/min.

**3-(4-(Difluoromethoxy)phenyl)-5-phenethoxy-8-(trifluoromethyl)-[1,2,4]triazolo[4,3-*a*]pyrazine (10).** White amorphous solid (6 mg, 12%); UV (MeOH) λ_max_ (log ε): 243 (3.94), 324 (3.50) nm; ^1^H NMR (800 MHz, CDCl_3_) δ_H_ 2.96 (t, *J* = 6.6 Hz, 2H, H-18), 4.54 (t, *J* = 6.6 Hz, 2H, H-17), 6.58 (t, *J* = 73.5 Hz, 1H, H-16), 6.86 (m, 2H, H-20, H-24), 7.19 (m, 2H, H-12, H-14), 7.22 (m, 1H, H-22), 7.23 (m, 2H, H-21, H-23), 7.37 (s, 1H, H-6), 7.62 (m, 2H, H-11, H-15); ^13^C NMR (200 MHz, CDCl_3_) δ_C_ 34.5 (C-18), 72.1 (C-17), 106.9 (C-6), 115.6 (t, *J* = 262.1 Hz, C-16), 118.9 (C-12, C-14), 120.2 (q, *J* = 273.3 Hz, C-25), 124.2 (C-10), 127.4 (C-22), 128.5 (C-20, C-24), 128.9 (C-21, C-23), 132.6 (C-11, C-15), 133.1 (q, *J* = 38.8 Hz, C-8), 135.8 (C-19), 144.2 (C-9), 145.8 (C-5), 147.2 (C-3), 152.7 (t, *J* = 2.7 Hz, C-13); LRESIMS *m/z*: 451 [M + H]^+^, 473 [M + Na]^+^, 923 [2M + Na]^+^; HRESIMS (*m/z*): [M + H]^+^ calcd for C_21_H_16_F_5_N_4_O_2_, 451.1188; found, 451.1195.

**8-(1,1-Difluoroethyl)-3-(4-(difluoromethoxy)phenyl)-5-phenethoxy-[1,2,4]triazolo[4,3-*a*]pyrazine (11).** Yellow amorphous solid (23 mg, 51%); UV (MeOH) λ_max_ (log ε): 240 (4.30), 317 (3.88) nm; ^1^H NMR (800 MHz, CDCl_3_) δ_H_ 2.27 (t, *J* = 18.7 Hz, 3H, H-26), 2.94 (t, *J* = 6.5 Hz, 2H, H-18), 4.48 (t, *J* = 6.5 Hz, 2H, H-17), 6.56 (t, *J* = 72.1 Hz, 1H, H-16), 6.86 (m, 2H, H-20, H-24), 7.18 (m, 2H, H-12, H-14), 7.22 (m, 1H, H-22), 7.23 (m, 2H, H-21, H-23), 7.30 (s, 1H, H-6), 7.62 (m, 2H, H-11, H-15); ^13^C NMR (200 MHz, CDCl_3_) δ_C_ 23.2 (t, *J* = 26.3 Hz, C-26), 34.5 (C-18), 71.7 (C-17), 106.6 (C-6), 115.6 (t, *J* = 260.3 Hz, C-16), 118.8 (C-12, C-14), 119.7 (t, *J* = 241.1 Hz, C-25), 124.7 (C-10), 127.3 (C-22), 128.6 (C-20, C-24), 128.9 (C-21, C-23), 132.6 (C-11, C-15), 140.2 (t, *J* = 31.3 Hz, C-8), 136.1 (C-19), 144.8 (C-9), 144.9 (C-5), 146.8 (C-3), 152.6 (t, *J* = 2.8 Hz, C-13); LRESIMS *m/z*: 447 [M + H]^+^, 915 [2M + Na]^+^; HRESIMS (*m/z*): [M + H]^+^ calcd for C_22_H_19_F_4_N_4_O_2_, 447.1439; found, 447.1457.

**3-(4-(Difluoromethoxy)phenyl)-8-(difluoromethyl)-5-phenethoxy-[1,2,4]triazolo[4,3-*a*]pyrazine (12).** White amorphous solid (14 mg, 31%); UV (MeOH) λ_max_ (log ε): 242 (4.16), 321 (3.73) nm; ^1^H NMR (800 MHz, CDCl_3_) δ_H_ 2.96 (t, *J* = 6.5 Hz, 2H, H-18), 4.52 (t, *J* = 6.5 Hz, 2H, H-17), 6.57 (t, *J* = 73.0 Hz, 1H, H-16), 6.86 (m, 2H, H-20, H-24), 7.09, (t, *J* = 54.0 Hz, 1H, H-25), 7.18 (m, 2H, H-12, H-14), 7.21 (m, 1H, H-22), 7.22 (m, 2H, H-21, H-23), 7.34 (s, 1H, H-6), 7.61 (m, 2H, H-11, H-15); ^13^C NMR (200 MHz, CDCl_3_) δ_C_ 34.4 (C-18), 71.9 (C-17), 107.5 (C-6), 115.6 (t, *J* = 262.2 Hz, C-16), 118.8 (C-12, C-14), 111.5 (t, *J* = 241.3 Hz, C-25), 124.4 (C-10), 127.2 (C-22), 128.5 (C-20, C-24), 128.8 (C-21, C-23), 132.6 (C-11, C-15), 137.5 (t, *J* = 26.4 Hz, C-8), 136.0 (C-19), 145.0 (C-9), 145.2 (C-5), 147.0 (C-3), 152.7 (t, *J* = 2.7 Hz, C-13); LRESIMS *m/z*: 433 [M + H]^+^, 887 [2M + Na]^+^; HRESIMS (*m/z*) [M + H]^+^ calcd for C_21_H_17_F_4_N_4_O_2_, 433.1282; found, 433.1282.

**3-(4-Chlorophenyl)-5-phenethoxy-8-(trifluoromethyl)-[1,2,4]triazolo[4,3-*a*]pyrazine (13).** White amorphous solid (3 mg, 6%); UV (MeOH) λ_max_ (log ε): 244 (3.69), 286 (3.19), 322 (3.22) nm; ^1^H NMR (800 MHz, CDCl_3_) δ_H_ 2.98 (t, *J* = 6.6 Hz, 2H, H-17), 4.55 (t, *J* = 6.6 Hz, 2H, H-16), 6.88 (m, 2H, H-19, H-23), 7.24 (m, 1H, H-21), 7.25 (m, 2H, H-20, H-22), 7.39 (s, 1H, H-6), 7.42 (m, 2H, H-12, H-14), 7.54 (m, 2H, H-11, H-15); ^13^C NMR (200 MHz, CDCl_3_) δ_C_ 34.5 (C-17), 72.2 (C-16), 107.0 (C-6), 120.0 (q, *J* = 274.4 Hz, C-24), 125.4 (C-10), 127.4 (C-21), 128.3 (C-12, C-14), 128.5 (C-19, C-23), 129.0 (C-20, C-22), 132.2 (C-11, C-15), 133.2 (q, *J* = 38.9 Hz, C-8), 135.7 (C-18), 137.1 (C-13), 144.1 (C-9), 145.8 (C-5), 147.2 (C-3); LRESIMS *m/z* 419 [M + H]^+^, 441 [M + Na]^+^, 859 [2M + Na]^+^; HRESIMS (*m/z*) [M + H]^+^ calcd for C_20_H_15_ClF_3_N_4_O, 419.0881; found, 419.0887.

**3-(4-Chlorophenyl)-8-(1,1-difluoroethyl)-5-phenethoxy-[1,2,4]triazolo[4,3-*a*]pyrazine (14).** Yellow amorphous solid (18 mg, 44%); UV (MeOH) λ_max_ (log ε): 243 (4.23), 286 (3.81), 317 (3.80) nm; ^1^H NMR (800 MHz, CDCl_3_) δ_H_ 2.26 (t, *J* = 18.9 Hz, 3H, H-25), 2.95 (t, *J* = 6.5 Hz, 2H, H-17), 4.49 (t, *J* = 6.5 Hz, 2H, H-16), 6.88 (m, 2H, H-19, H-23), 7.23 (m, 1H, H-21), 7.24 (m, 2H, H-20, H-22), 7.30 (s, 1H, H-6), 7.40 (m, 2H, H-12, H-14), 7.53 (m, 2H, H-11, H-15); ^13^C NMR (200 MHz, CDCl_3_) δ_C_ 23.1 (t, *J* = 26.2 Hz, C-25), 34.5 (C-17), 71.7 (C-16), 106.7 (C-6), 119.7 (t, *J* = 240.3 Hz, C-24), 126.0 (C-10), 127.3 (C-21), 128.2 (C-12, C-14), 128.6 (C-19, C-23), 128.9 (C-20, C-22), 132.2 (C-11, C-15), 136.0 (C-18), 136.7 (C-13), 140.2 (t, *J* = 31.7 Hz, C-8), 144.8 (C-5), 144.9 (C-9), 146.8 (C-3); LRESIMS *m/z*: 415 [M + H]^+^, 851 [2M + Na]^+^; HRESIMS (*m/z*): [M + H]^+^ calcd for C_21_H_18_ClF_2_N_4_O, 415.1132, found, 415.1141.

**3-(4-Chlorophenyl)-8-(difluoromethyl)-5-phenethoxy-[1,2,4]triazolo[4,3-*a*]pyrazine (15).** White amorphous solid (11 mg, 28%); UV (MeOH) λ_max_ (log ε): 244 (4.26), 286 (3.82), 319 (3.83) nm; ^1^H NMR (800 MHz, CDCl_3_) δ_H_ 2.97 (t, *J* = 6.5 Hz, 2H, H-17), 4.54 (t, *J* = 6.5 Hz, 2H, H-16), 6.87 (m, 2H, H-19, H-23), 7.05 (t, *J* = 53.5 Hz, 1H, H-24), 7.22 (m, 1H, H-21), 7.23 (m, 2H, H-20, H-22), 7.40 (s, 1H, H-6), 7.40 (m, 2H, H-12, H-14), 7.52 (m, 2H, H-11, H-15); ^13^C NMR (200 MHz, CDCl_3_) δ_C_ 34.4 (C-17), 72.1 (C-16), 107.8 (C-6), 111.6 (t, *J* = 241.8 Hz, C-24), 125.1 (C-10), 127.3 (C-21), 128.3 (C-12, C-14), 128.5 (C-19, C-23), 128.9 (C-20, C-22), 132.1 (C-11, C-15), 135.8 (C-18), 137.1 (C-13), 137.3 (t, *J* = 26.6 Hz, C-8), 144.5 (C-9), 145.2 (C-5), 147.0 (C-3); LRESIMS *m/z*: 401 [M + H]^+^, 823 [2M + Na]^+^; HRESIMS (*m/z*): [M + H]^+^ calcd for C_20_H_16_ClF_2_N_4_O, 401.0975; found, 401.0978.

**4-(5-Phenethoxy-8-(trifluoromethyl)-[1,2,4]triazolo[4,3-*a*]pyrazin-3-yl)benzonitrile (16).** White amorphous solid (5 mg, 11%); UV (MeOH) λ_max_ (log ε): 226 (4.26), 249 (4.20), 297 (3.95) nm; ^1^H NMR (800 MHz, CDCl_3_) δ_H_ 2.99 (t, *J* = 6.5 Hz, 2H, H-18), 4.62 (t, *J* = 6.5 Hz, 2H, H-17), 6.90 (m, 2H, H-20, H-24), 7.26 (m, 1H, H-22), 7.27 (m, 2H, H-21, H-23), 7.47 (s, 1H, H-6), 7.64 (m, 2H, H-12, H-14), 7.68 (m, 2H, H-11, H-15); ^13^C NMR (200 MHz, CDCl_3_) δ_C_ 34.2 (C-18), 72.1 (C-17), 107.6 (C-6), 114.3 (C-13), 118.1 (C-16), 120.0 (q, *J* = 274.6 Hz, C-25), 127.6 (C-22), 128.3 (C-20, C-24), 129.1 (C-21, C-23), 131.1 (C-10), 131.5 (C-11, C-15), 131.6 (C-12, C-14), 133.1 (q, *J* = 39.1 Hz, C-8), 135.4 (C-19), 144.1 (C-9), 145.6 (C-5), 146.4 (C-3); LRESIMS *m/z*: 410 [M + H]^+^, 432 [M + Na]^+^; HRESIMS (*m/z*): [M + H]^+^ calcd for C_21_H_15_F_3_N_5_O, 410.1223; found, 410.1221.

**4-(8-(1,1-Difluoroethyl)-5-phenethoxy-[1,2,4]triazolo[4,3-*a*]pyrazin-3-yl)benzonitrile (17).** Yellow amorphous solid (23 mg, 56%); UV (MeOH) λ_max_ (log ε): 226 (4.41), 250 (4.33), 294 (4.15) nm; ^1^H NMR (800 MHz, CDCl_3_) δ_H_ 2.27 (t, *J* = 18.7 Hz, 3H, H-26), 2.97 (t, *J* = 6.5 Hz, 2H, H-18), 4.55 (t, *J* = 6.5 Hz, 2H, H-17), 6.90 (m, 2H, H-20, H-24), 7.27 (m, 1H, H-22), 7.27 (m, 2H, H-21, H-23), 7.38 (s, 1H, H-6), 7.64 (m, 2H, H-12, H-14), 7.69 (m, 2H, H-11, H-15); ^13^C NMR (200 MHz, CDCl_3_) δ_C_ 23.1 (t, *J* = 26.4 Hz, C-26), 34.3 (C-18), 71.5 (C-17), 107.0 (C-6), 114.0 (C-13), 118.3 (C-16), 119.7 (t, *J* = 240.3 Hz, C-25), 127.5 (C-22), 128.4 (C-20, C-24), 129.0 (C-21, C-23), 131.46 (C-12, C-14), 131.51 (C-11, C-15), 131.9 (C-10), 135.7 (C-19), 140.4 (t, *J* = 31.7 Hz, C-8), 144.7 (C-5), 145.0 (C-9), 146.1 (C-3); LRESIMS *m/z*: 406 [M + H]^+^, 428 [M + Na]^+^; HRESIMS (*m/z*): [M + H]^+^ calcd for C_22_H_18_F_2_N_5_O, 406.1474; found, 406.1473.

**4-(8-(Difluoromethyl)-5-phenethoxy-[1,2,4]triazolo[4,3-*a*]pyrazin-3-yl)benzonitrile (18).** White crystalline solid (11 mg, 27%); mp 109–111 °C; UV (MeOH) λ_max_ (log ε): 227 (4.56), 248 (4.48), 299 (4.28) nm; ^1^H NMR (800 MHz, CDCl_3_) δ_H_ 2.99 (t, *J* = 6.5 Hz, 2H, H-18), 4.60 (t, *J* = 6.5 Hz, 2H, H-17), 6.90 (m, 2H, H-20, H-24), 7.06, (t, *J* = 53.4 Hz, 1H, H-25), 7.26 (m, 1H, H-22), 7.26 (m, 2H, H-21, H-23), 7.49 (s, 1H, H-6), 7.64 (m, 2H, H-12, H-14), 7.68 (m, 2H, H-11, H-15); ^13^C NMR (200 MHz, CDCl_3_) δ_C_ 34.3 (C-18), 71.9 (C-17), 108.2 (C-6), 111.6 (t, *J* = 241.7 Hz, C-25), 114.3 (C-13), 118.0 (C-16), 127.5 (C-22), 128.3 (C-20, C-24), 129.0 (C-21, C-23), 131.0 (C-10), 131.5 (C-11, C-15), 131.6 (C-12, C-14), 135.5 (C-19), 137.5 (t, *J* = 27.3 Hz, C-8), 144.6 (C-9), 145.0 (C-5), 146.2 (C-3); LRESIMS *m/z*: 392 [M + H]^+^, 414 [M + Na]^+^, 805 [2M + Na]^+^; HRESIMS (*m/z*): [M + H]^+^ calcd for C_21_H_16_F_2_N_5_O, 392.1317; found, 392.1317.

## Supporting Information

File 1 General experimental procedures, NMR spectra and characterisation data for all new triazolopyrazine compounds and X-ray crystallography data for compounds **5**, **6** and **18**.
