# Supplementary material for: Synthesis and characterisation of new antimalarial fluorinated triazolopyrazine compounds
Source: Beilstein J Org Chem. 2023 Jan 31;19:107–14. doi: 10.3762/bjoc.19.11 (PMC9907012; doi:10.3762/bjoc.19.11)
Supplement: File 1 — General experimental procedures, NMR spectra and characterisation data for all new triazolopyrazine compounds and X-ray crystallography data for compounds 5, 6 and 18. [file Beilstein_J_Org_Chem-19-107-s001.pdf]

BEILSTEIN JOURNAL OF ORGANIC CHEMISTRY

Supporting Information

for

Synthesis and characterisation of new antimalarial fluorinated triazolopyrazine compounds

Kah Yean Lum, Jonathan M. White, Daniel J. G. Johnson, Vicky M. Avery
and Rohan A. Davis

Beilstein J. Org. Chem. **2023,** *19,* 107–114. doi:10.3762/bjoc.19.11

General experimental procedures, NMR spectra and characterisation data for all new triazolopyrazine compounds and X-ray crystallography data for compounds 5, 6 and 18

License and Terms: This is a supporting information file under the terms of the Creative Commons Attribution License (https://creativecommons.org/licenses/by/4.0). Please note that the reuse, redistribution and reproduction in particular requires that the author(s) and source are credited and that individual graphics may be subject to special legal provisions.
The license is subject to the *Beilstein Journal of Organic Chemistry* terms and conditions: (https://www.beilstein-journals.org/bjoc/terms)

Table of contents

S1: General experimental procedures. S3
S2: General procedure for coupling of primary alcohols with chloro-heterocycle scaffolds. S3
S3: X-ray crystallography analysis S3
S4: In vitro anti-plasmodial image-based assay S4
S5: In vitro cytotoxicity assay S4
S6: *3-(4-(difluoromethoxy)phenyl)-5-phenethoxy-[1,2,4]triazolo[4,3-a]pyrazine* (**4**) [6] S5
S7: ^{1}H NMR spectrum of compound **4** in $CDCl_3$ S6
S8: ^{13}C NMR spectrum of compound **4** in $CDCl_3$[a] S6
S9: *3-(4-chlorophenyl)-5-phenethoxy-[1,2,4]triazolo[4,3-a]pyrazine* (**5**) S7
S10: ^{1}H NMR spectrum of compound **5** in $CDCl_3$ S7
S11: ^{13}C NMR spectrum of compound **5** in $CDCl_3$ S8
S12: *4-(5-phenethoxy-[1,2,4]triazolo[4,3-a]pyrazin-3-yl)benzonitrile* (**6**) S8
S13: ^{1}H NMR spectrum of compound **6** in $CDCl_3$ S9
S14: ^{13}C NMR spectrum of compound **6** in $CDCl_3$ S9
S15: *3-(4-(difluoromethoxy)phenyl)-5-ethoxy-[1,2,4]triazolo[4,3-a]pyrazine* (**7**) S10
S16: ^{1}H NMR spectrum of compound **7** in $CDCl_3$ S10
S17: ^{13}C NMR spectrum of compound **7** in $CDCl_3$ S11
S18: *3-(4-chlorophenyl)-5-ethoxy-[1,2,4]triazolo[4,3-a]pyrazine* (**8**) S11
S19: ^{1}H NMR spectrum of compound **8** in $CDCl_3$ S12
S20: ^{13}C NMR spectrum of compound **8** in $CDCl_3$ S12
S21: *4-(5-ethoxy-[1,2,4]triazolo[4,3-a]pyrazin-3-yl)benzonitrile* (**9**) S13
S22: ^{1}H NMR spectrum of compound **9** in $CDCl_3$ S13
S23: ^{13}C NMR spectrum of compound **9** in $CDCl_3$ S14
S24: NMR data of compound **10** in $CDCl_3$[a] S15
S25: ^{1}H NMR spectrum of compound **10** in $CDCl_3$ S15
S26: ^{13}C NMR spectrum of compound **10** in $CDCl_3$ S16
S27: NMR data of compound **11** in $CDCl_3$[a] S16
S28: ^{1}H NMR spectrum of compound **11** in $CDCl_3$ S17
S29: ^{13}C NMR spectrum of compound **11** in $CDCl_3$ S17
S30: NMR data of compound **12** in $CDCl_3$[a] S18
S31: ^{1}H NMR spectrum of compound **12** in $CDCl_3$ S18
S32: ^{13}C NMR spectrum of compound **12** in $CDCl_3$ S19
S33: NMR data of compound **13** in $CDCl_3$[a] S19
S34: ^{1}H NMR spectrum of compound **13** in $CDCl_3$ S20

S35: ^{13}C NMR spectrum of compound **13** in $CDCl_3$ S20
S36: NMR data of compound **14** in $CDCl_3$[a] S21
S37: ^{1}H NMR spectrum of compound **14** in $CDCl_3$ S21
S38: ^{13}C NMR spectrum of compound **14** in $CDCl_3$ S22
S39: NMR data of compound **15** in $CDCl_3$[a] S22
S40: ^{1}H NMR spectrum of compound **15** in $CDCl_3$ S23
S41: ^{13}C NMR spectrum of compound **15** in $CDCl_3$ S23
S42: NMR data of compound **16** in $CDCl_3$[a] S24
S43: ^{1}H NMR spectrum of compound **16** in $CDCl_3$ S24
S44: ^{13}C NMR spectrum of compound **16** in $CDCl_3$ S25
S45: NMR data of compound **17** in $CDCl_3$[a] S25
S46: ^{1}H NMR spectrum of compound **17** in $CDCl_3$ S26
S47: ^{13}C NMR spectrum of compound **17** in $CDCl_3$ S26
S48: NMR data of compound **18** in $CDCl_3$[a] S27
S49: ^{1}H NMR spectrum of compound **18** in $CDCl_3$ S27
S50: ^{13}C NMR spectrum of compound **18** in $CDCl_3$ S28
S51: Crystal data for compound **5** S28
S52: Crystal data for compound **6** S28
S53: Crystal data for compound **18** S29
S54: ORTEP drawing of compounds **5** and **6** S29

S1: General experimental procedures

Melting points were measured using a Cole-Parmer melting point apparatus and are uncorrected. UV spectra were recorded using an Ocean Optics (USB-ISS-UV/VIS) spectrophotometer. NMR spectra were recorded at 25 °C on a Bruker AVANCE III HD 800 MHz NMR spectrometer equipped with a cryoprobe. The ^{1}H and ^{13}C chemical shifts were referenced to solvent peaks for $CDCl_3$ at δ_H 7.26 and δ_C 77.16, respectively. LRESIMS data were recorded on an Ultimate 3000 RS UHPLC coupled to a Thermo Fisher Scientific MSQ Plus single quadruple ESI mass spectrometer. HRESIMS data were acquired on a Bruker SolariX XR 12T FT-ICR-MS. GRACE Davisil C_{18}-bonded silica (35–70 µm, 60 Å) was used for pre-adsorption work before HPLC separations, and the pre-absorbed sample was packed into an Alltech stainless steel guard cartridge (10 × 30 mm). A Thermo Fisher Scientific Dionex Ultimate 3000 UHPLC was used for semipreparative HPLC separations. A Thermo Electron Betasil C_{18}-bonded silica column (5 µm, 100 Å, 150 × 21.2 mm) and a Thermo Betasil phenyl-bonded silica column (5 µm, 100 Å, 150 × 21.2 mm) were used for semipreparative HPLC separations. All solvents used for chromatography, UV and MS were Honeywell Burdick & Jackson or Lab-Scan HPLC grade. H_2O was filtered using a Sartorius Stedium Arium Pro VF ultrapure water system. All chemical reagents were purchased from Sigma-Aldrich.

S2: General procedure for coupling of primary alcohols with chloro-heterocycle scaffolds

The chloro-heterocycle scaffold (0.4 mmol), primary alcohol (0.4 mmol, 1.0 eq.), KOH (67 mg, 1.2 mmol, 3.0 eq.) and 18-crown-6 (8 mg, 32 µmol, 0.08 eq.) were dissolved in anhydrous acetonitrile (2 mL) and the reaction mixture stirred at room temperature for 2 h then preadsorbed to silica (~1 g) overnight. Purification was performed on an Isolute (10 g, silica, 55 µm, 54 Å) SPE cartridge using a 2% stepwise elution from 100% CH_2Cl_2 to 6% MeOH/94% CH_2Cl_2 with five fractions (10 mL each) collected for each elution. The 20 resulting fractions were all analysed by silica TLC (solvent system: 5% MeOH/96% CH_2Cl_2) and UV-active (254 nm) samples were further evaluated by ^{1}H NMR spectroscopy and LCMS in order to identify product of interest; only fractions containing the desired product in high purity (>95%) were combined. All ether triazolopyrazines eluted from the silica SPE cartridge with 4% MeOH/96% CH_2Cl_2 in yields ranging from 54 to 78%.

S3: X-ray crystallography analysis

Intensity data for compounds **5**, **6**, **18** were collected with an Oxford Diffraction Synergy diffractometer with Cu Kα radiation, and the temperature during data collection was maintained at 100.0(1) K using an Oxford Cryosystems cooling device. The structure of each

compound was solved by direct methods and difference Fourier synthesis [1]. Hydrogen atoms were placed in their idealized positions and included in subsequent refinement cycles. Thermal ellipsoid plots were generated in Mercury [2] within the WINGX suite of programs [3]. Crystallographic data for compounds **5** (CCDC 2195529), **6** (CCDC 2195531) and **18** (CCDC 2195530) have been deposited with the Cambridge Crystallographic Data Centre. These data can be obtained free of charge from the Cambridge Crystallographic Data Centre via http://www.ccdc.cam.ac.uk/data_request/cif.

S4: In vitro anti-plasmodial image-based assay

Plasmodium falciparum 3D7 and Dd2 parasites were cultured in RPMI1640 (Life Technologies, Camarillo, CA, USA) supplemented with 2.5 mg/mL Albumax II, 5% AB human serum, 25 mM HEPES, and 0.37mM hypoxanthine. Ring stage parasites were treated with compounds following two rounds of sorbitol synchronization, as previously described [4]. Artesunate and pyrimethamine were incorporated as controls. Following incubation of assay plates for 72 h at 37 °C, and 5% CO_2 and 5% O_2, parasites were stained with 2-(4-amidinophenyl)-1H-indole-6-carboxamidine (DAPI) and imaged using an Opera Phenix™ High Content Screening System (PerkinElmer, Waltham, MA, USA). Images were analysed using Harmony software (PerkinElmer, Waltham, MA, USA).

S5: In vitro cytotoxicity assay

Human embryonic kidney (HEK293) cells were maintained in DMEM (Life Technologies, Camarillo, CA, USA) containing 10% FBS (Hyclone™ ThermoFisher, Melbourne, Australia). Cytotoxicity testing was undertaken, as previously described [5]. In brief, 5 μL of test compound was added to the well of black/clear tissue culture-treated, 384-well plates containing 3000 adherent HEK 293 cells/well in 45 μL and incubated 72 h at 37 °C in 5% CO_2. After incubation, the supernatant was removed and replaced with 40 μL of 10% Alamar Blue per well. Plates were incubated for 5−6 h and measured for fluorescence at 530-nm excitation and 595-nm emission. The % inhibition was calculated using 0.4% DMSO (no inhibition) and 5 μM puromycin (100% inhibition) data. IC_{50} values were obtained by plotting % inhibition against log dose using GraphPad Prism v.6 (San Diego, CA, USA) nonlinear regression with a variable slope plot.

S6: *3-(4-(difluoromethoxy)phenyl)-5-phenethoxy-[1,2,4]triazolo[4,3-a]pyrazine* (**4**) [6]

White amorphous solid (119 mg, 78%); UV (MeOH) λ_{max} (log ε) 240 (4.40), 285 (4.01), 307 (3.99) nm; LRESIMS *m/z* 383 $[M + H]^+$; HRESIMS *m/z* 383.1318 $[M + H]^+$ (calcd for $C_{20}H_{17}F_2N_4O_2$, 383.1314).

Table S1: NMR data of compound **4** in $CDCl_3$[a]

**Position**	**δ_H, mult. (*J* in Hz), int.**	**δ_C, mult. (*J* in Hz)**	**COSY**	**HMBC**	**ROESY**
3		146.4, s			
5		144.0, s			
6	7.32, s, 1H	108.4, s		5, 8	17
8	9.02, s, 1H	136.6, s		5, 6, 9	
9		147.9, s			
10		125.0, s			
11	7.65, m, 1H	132.5, s	12	3, 13, 15	12
12	7.18, m, 1H	118.8, s	11	10, 14	11
13		152.5, t (2.7)			
14	7.18, m, 1H	118.8, s	15	10, 12	15
15	7.65, m, 1H	132.5, s	14	3, 11, 13	14
16	6.57, t (73.1), 1H	115.7, t (261.1)		13	
17	4.45, t (6.5), 2H	71.3, s	18	5, 18, 19	6, 18
18	2.96, t (6.5), 2H	34.6, s	17	17, 19, 20, 24	17, 20, 24
19		136.3, s			
20	6.88, m, 1H	128.6, s	21	18, 22, 24	18, 21
21	7.23, m, 1H	128.9, s	20	19, 23	20
22	7.22, m, 1H	127.2, s		20, 24	
23	7.23, m, 1H	128.9, s	24	19, 21	24
24	6.88, m, 1H	128.6, s	23	18, 20, 22	18, 23

[a]Recorded at 800 MHz (^1H NMR) and 200 MHz (^{13}C NMR) at 25 °C.

S7: ^{1}H NMR spectrum of compound **4** in $CDCl_3$

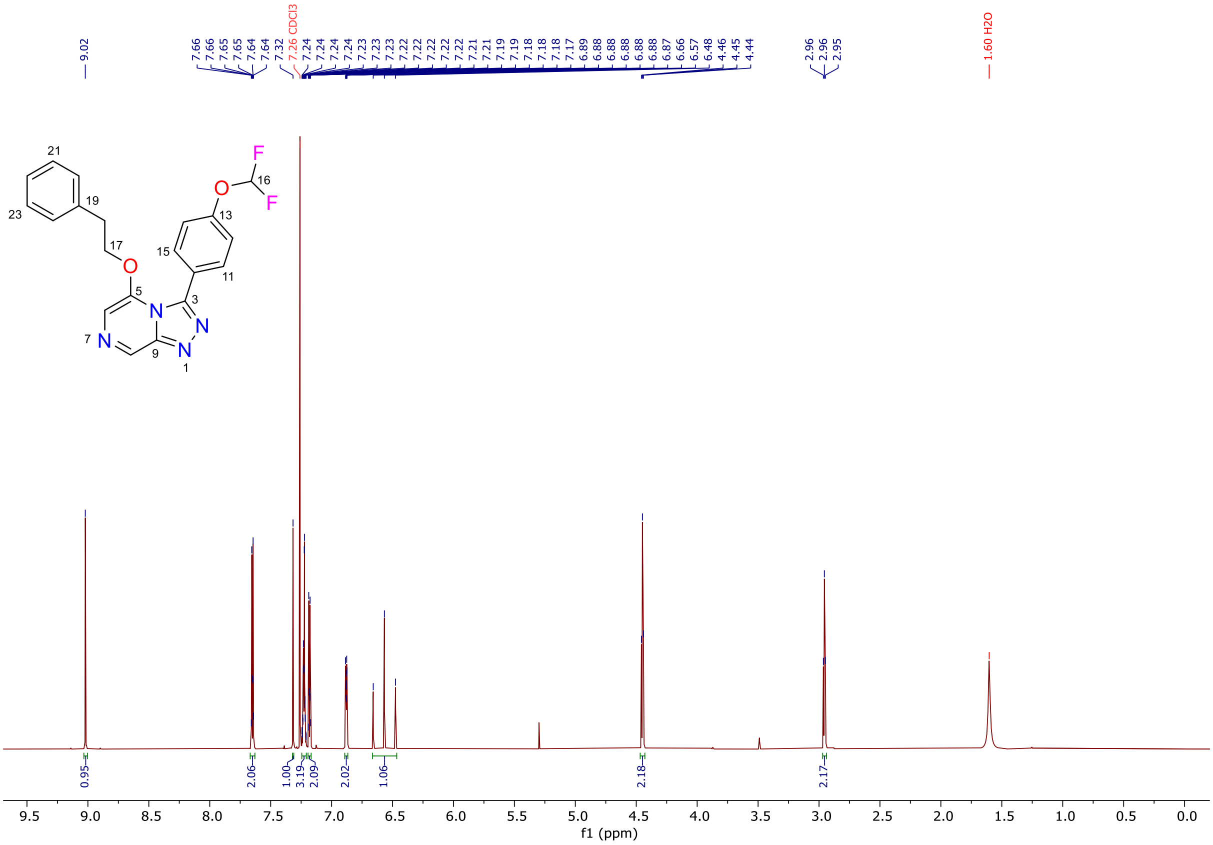

S8: ^{13}C NMR spectrum of compound **4** in $CDCl_3$[a]

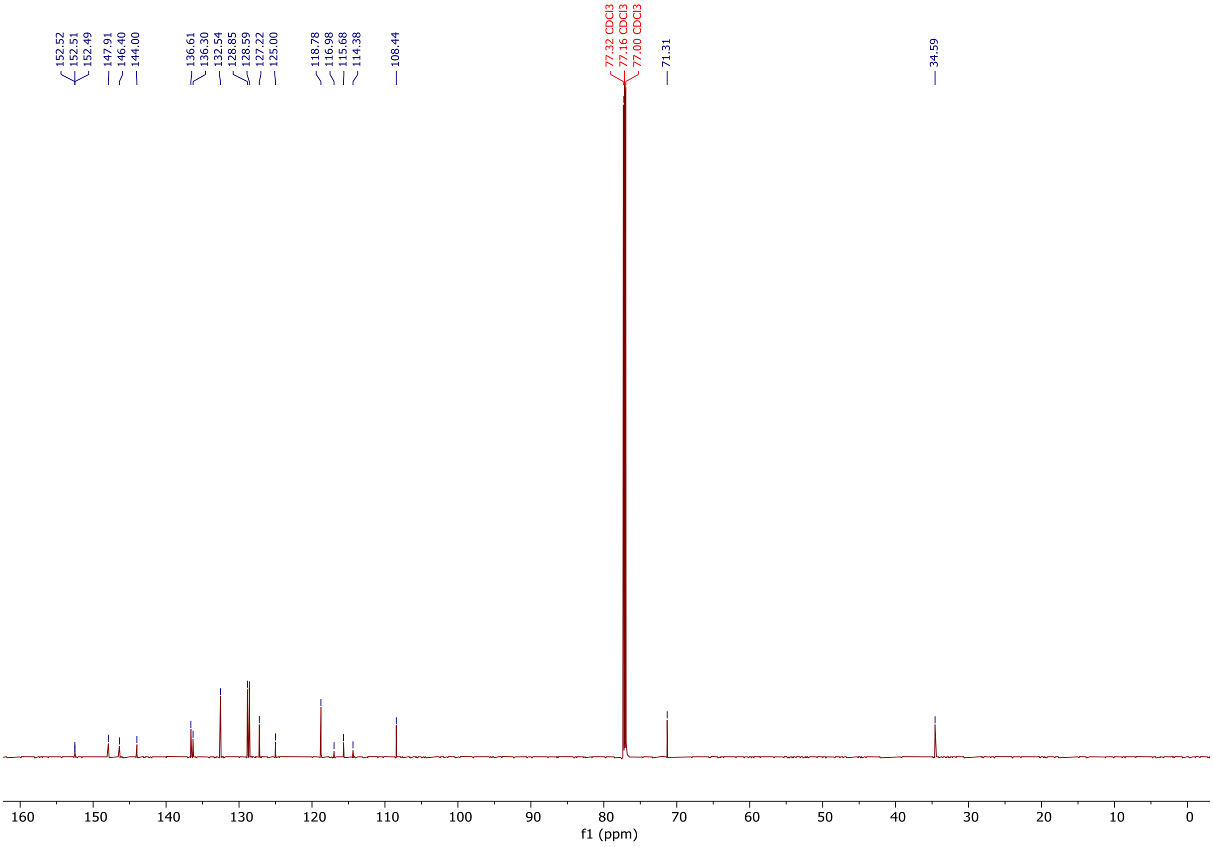

S9: *3-(4-chlorophenyl)-5-phenethoxy-[1,2,4]triazolo[4,3-a]pyrazine* (**5**)

Yellow amorphous solid (107 mg, 75%); mp 131–132 °C; UV (MeOH) λ_{max} (log ε) 242 (4.30), 290 (3.96) nm; LRESIMS *m/z* 351 $[M + H]^+$; HRESIMS *m/z* 351.1007 $[M + H]^+$ (calcd for $C_{19}H_{16}ClN_4O$, 351.1007).

Table S2: NMR data of compound **5** in $CDCl_3$[a]

Position	δ_H, mult. (*J* in Hz), int.	δ_C, mult. (*J* in Hz)	COSY	HMBC	ROESY
3		146.4, s			
5		144.0, s			
6	7.32, s, 1H	108.5, s		5, 8	16
8	9.01, s, 1H	136.52, s		5, 6, 9	
9		147.9, s			
10		126.3, s			
11	7.56, m, 1H	132.1, s	12	3, 13, 15	12
12	7.40, m, 1H	128.2, s	11	10, 14	11
13		136.51, s			
14	7.40, m, 1H	128.2, s	15	10, 12	15
15	7.56, m, 1H	132.1, s	14	3, 11, 13	14
16	4.45, t (6.5), 2H	71.3, s	17	5, 17, 18	6, 17
17	2.96, t (6.5), 2H	34.6, s	16	16, 18, 19, 23	16, 19, 23
18		136.2, s			
19	6.89, m, 1H	128.6, s	20	17, 21, 23	16, 17, 20
20	7.24, m, 1H	128.9, s	19	18, 22	19
21	7.23, m, 1H	127.2, s		19, 23	
22	7.24, m, 1H	128.9, s	23	18, 20	23
23	6.89, m, 1H	128.6, s	22	17, 19, 21	16, 17, 22

[a]Recorded at 800 MHz (^1H NMR) and 200 MHz (^{13}C NMR) at 25 °C.

S10: ^1H NMR spectrum of compound **5** in $CDCl_3$

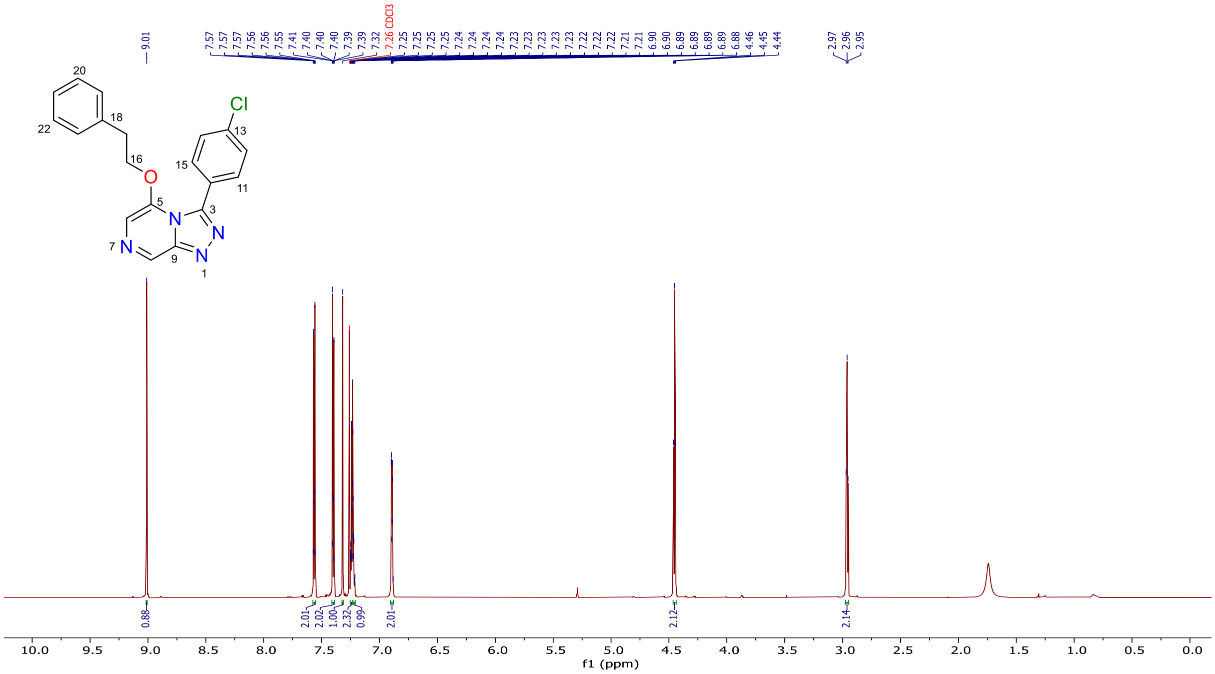

S11: ^{13}C NMR spectrum of compound **5** in $CDCl_3$

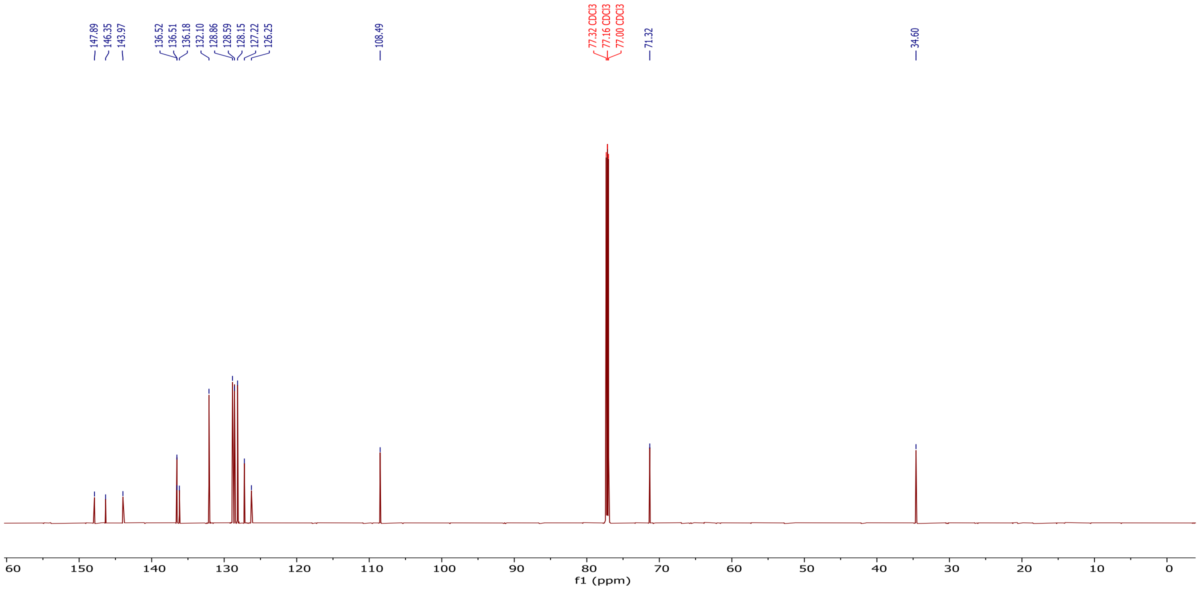

S12: *4-(5-phenethoxy-[1,2,4]triazolo[4,3-a]pyrazin-3-yl)benzonitrile* (**6**)

White amorphous solid (74 mg, 54%); mp 136–138 °C; UV (MeOH) λ_{max} (log ε) 261 (4.34), 311 (3.76) nm; LRESIMS *m/z* 342 $[M + H]^+$; HRESIMS *m/z* 342.1348 $[M + H]^+$ (calcd for $C_{20}H_{16}N_5O$, 342.1349).

Table S3: NMR data of compound **6** in $CDCl_3$[a]

Position	δ_H, mult. (*J* in Hz), int.	δ_C, mult. (*J* in Hz)	COSY	HMBC	ROESY
3		145.8, s			
5		144.2, s			
6	7.46, s, 1H	108.7, s		5, 8	17
8	9.09, s, 1H	135.8, s		5, 6, 9	
9		147.3, s			
10		131.52, s			
11	7.73, m, 1H	131.46, s	12	3, 13, 15	
12	7.63, m, 1H	131.52, s	11	10, 14, 16	
13		114.0, s			
14	7.63, m, 1H	131.52, s	15	10, 12, 16	
15	7.73, m, 1H	131.46, s	14	3, 11, 13	
16		118.2, s			
17	4.55, t (6.5), 2H	71.5, s	18	5, 18, 19	6, 18, 20, 24
18	2.98, t (6.5), 2H	34.4, s	17	17, 19, 20, 24	17, 20, 24
19		135.8, s			
20	6.91, m, 1H	128.4, s	21	18, 22, 24	17,18, 21
21	7.26, m, 1H	129.0, s	20	19, 23	
22	7.25, m, 1H	127.4, s		20, 24	
23	7.26, m, 1H	129.0, s	24	19, 21	
24	6.91, m, 1H	128.4, s	23	18, 20, 22	17, 18, 23

[a]Recorded at 800 MHz (^{1}H NMR) and 200 MHz (^{13}C NMR) at 25 °C.

S13: ^{1}H NMR spectrum of compound **6** in $CDCl_3$

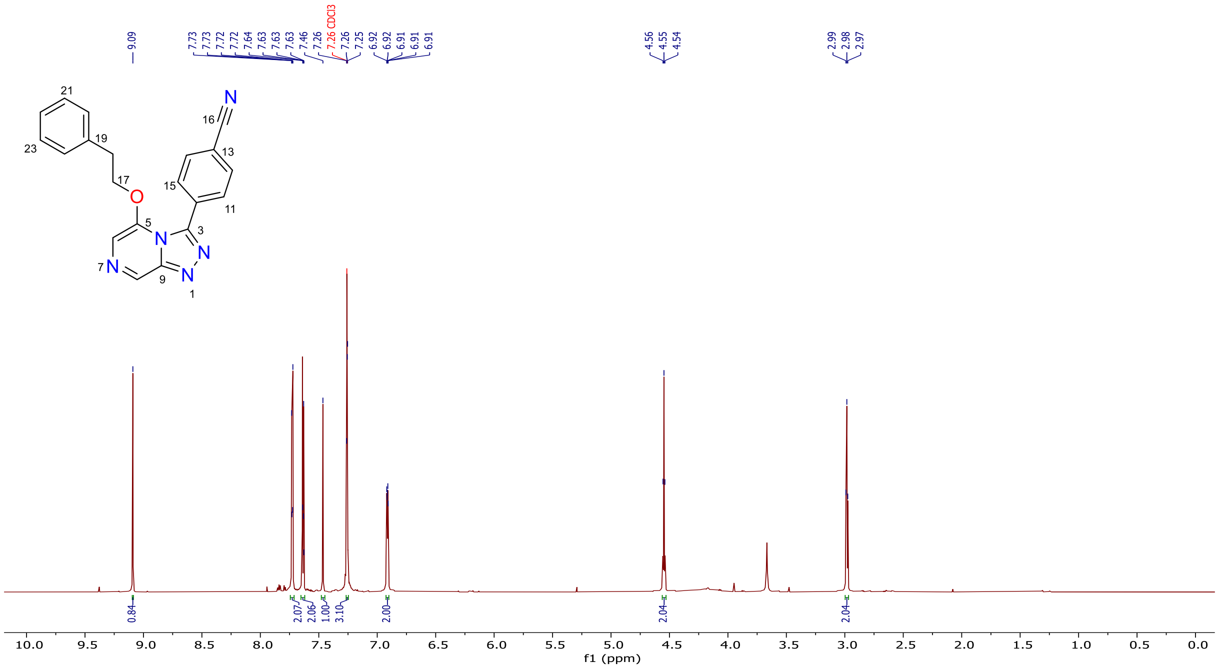

S14: ^{13}C NMR spectrum of compound **6** in $CDCl_3$

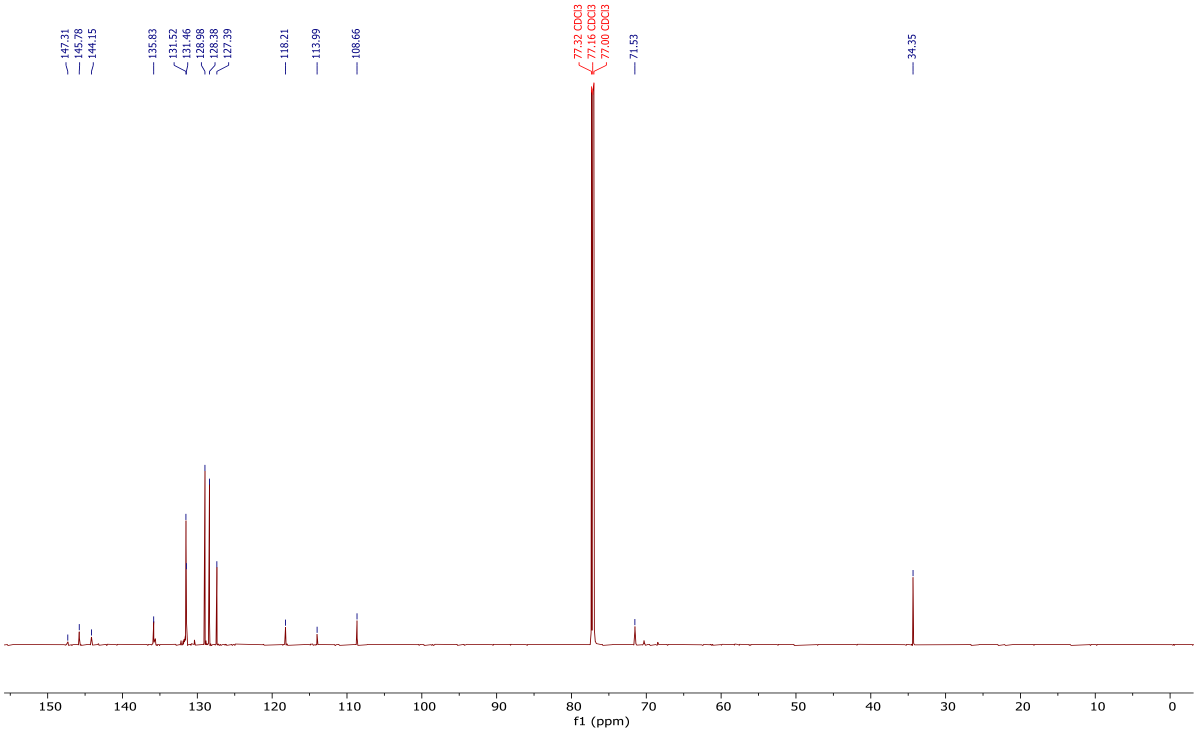

S15: *3-(4-(difluoromethoxy)phenyl)-5-ethoxy-[1,2,4]triazolo[4,3-a]pyrazine* (**7**)

Yellow amorphous solid (72 mg, 58%); UV (MeOH) λ_{max} (log ε) 240 (4.21), 285 (3.83), 307 (3.78) nm; LRESIMS *m/z* 307 $[M + H]^+$; HRESIMS *m/z* 307.1000 $[M + H]^+$ (calcd for $C_{14}H_{13}F_2N_4O_2$, 307.1001).

Table S4: NMR data of compound **7** in $CDCl_3$[a]

Position	δ_H, mult. (*J* in Hz), int.	δ_C, mult. (*J* in Hz)	COSY	HMBC	ROESY
3		146.6, s			
5		144.4, s			
6	7.35, s, 1H	108.2, s		5, 8	17
8	9.06, s, 1H	135.5, s		5, 6, 9	
9		147.1, s			
10		124.4, s			
11	7.73, m, 1H	132.7, s	12	3, 13, 15	12
12	7.24, m, 1H	118.7, s	11	10, 14	11
13		152.7, t (2.5)			
14	7.24, m, 1H	118.7, s	15	10, 12	15
15	7.73, m, 1H	132.7, s	14	3, 11, 13	14
16	6.61, t (73.6), 1H	115.7, t (262.2)		13	
17	4.30, q (7.0), 2H	67.4, s	18	5, 18	6, 18
18	1.29, t (7.0), 3H	14.0, s	17	17	17

[a]Recorded at 800 MHz (^{1}H NMR) and 200 MHz (^{13}C NMR) at 25 °C.

S16: ^{1}H NMR spectrum of compound **7** in $CDCl_3$

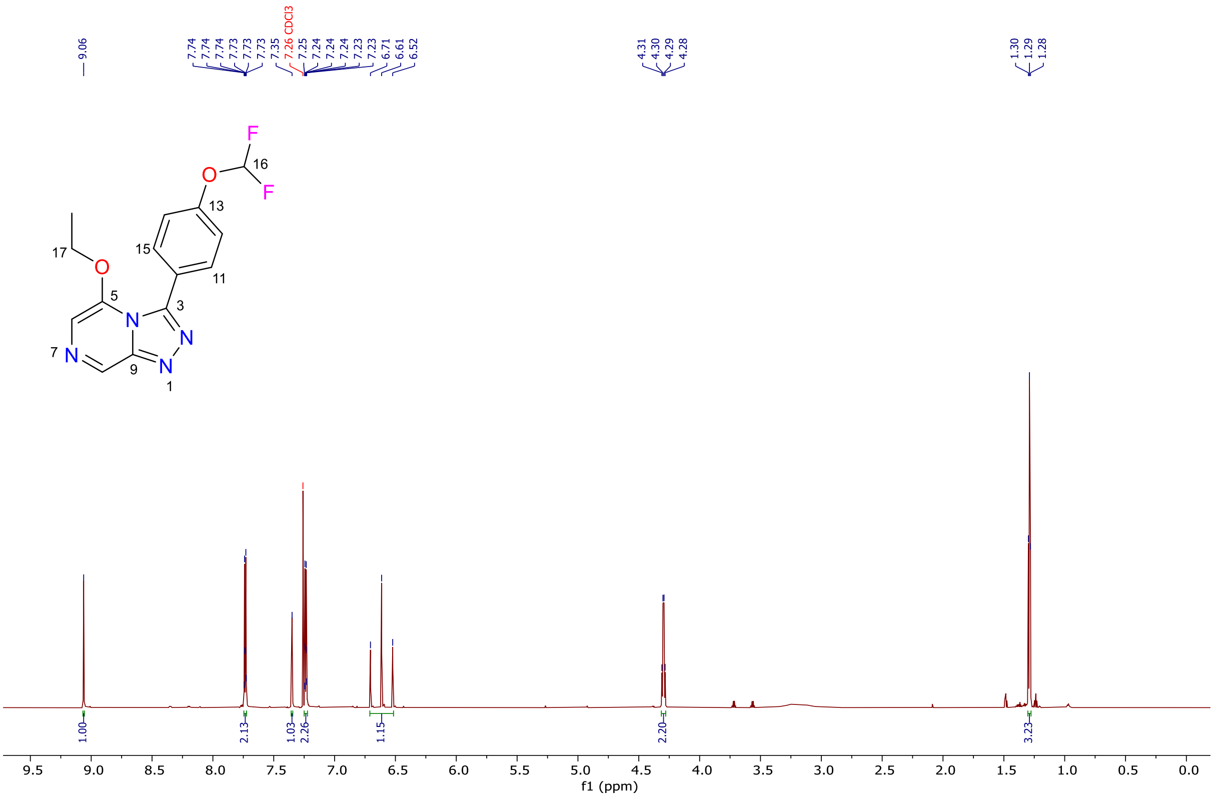

S17: ^{13}C NMR spectrum of compound **7** in $CDCl_3$

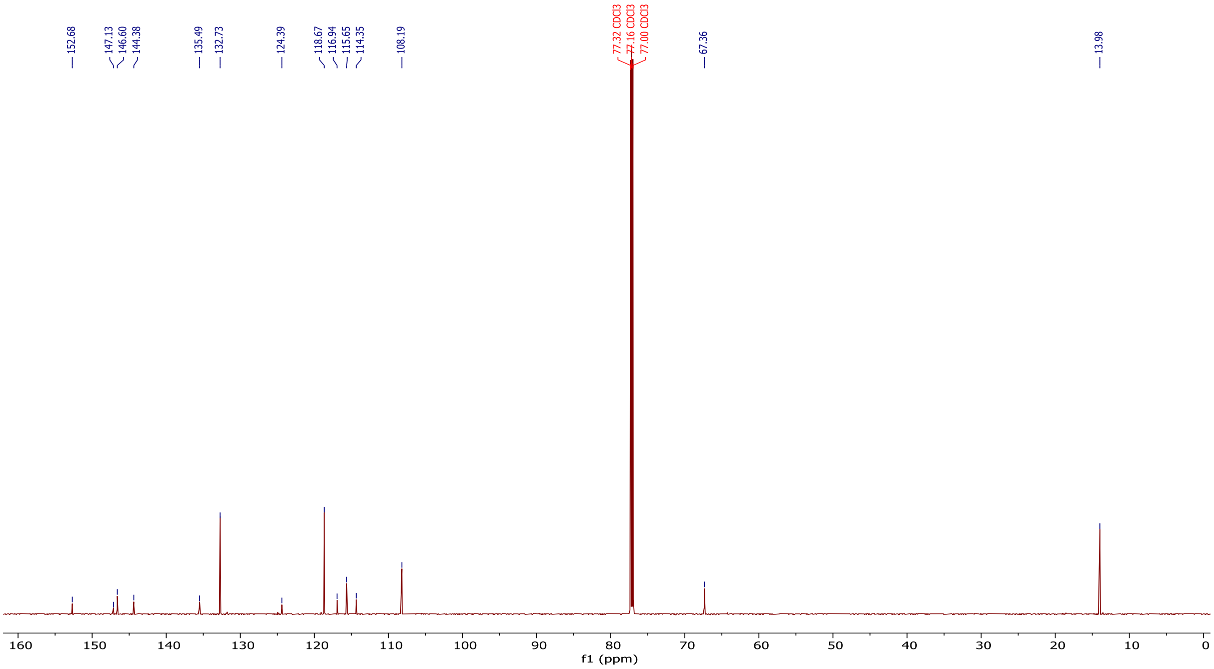

S18: *3-(4-chlorophenyl)-5-ethoxy-[1,2,4]triazolo[4,3-a]pyrazine* (**8**)

Pale yellow amorphous solid (35 mg, 64%); UV (MeOH) λ_{max} (log ε) 242 (4.12), 286 (3.76), 310 (3.70) nm; LRESIMS *m/z* 275 $[M + H]^+$; HRESIMS *m/z* 275.0698 $[M + H]^+$ (calcd for $C_{13}H_{12}ClN_4O$, 275.0694).

Table S5: NMR data of compound **8** in $CDCl_3$[a]

Position	δ$_H$, mult. (*J* in Hz), int.	δ$_C$, mult. (*J* in Hz)	COSY	HMBC	ROESY
3		146.6, s			
5		144.4, s			
6	7.38, s, 1H	108.2, s		5, 8	16
8	9.07, s, 1H	135.3, s		5, 6, 9	
9		147.0, s			
10		125.7, s			
11	7.68, m, 1H	132.3, s	12	3, 13, 15	12
12	7.47, m, 1H	128.1, s	11	10, 14	11
13		136.8, s			
14	7.47, m, 1H	128.1, s	15	10, 12	15
15	7.68, m, 1H	132.3, s	14	3, 11, 13	14
16	4.33, q (7.0), 2H	67.5, s	17	5, 17	6, 17
17	1.31, t (7.0), 3H	14.0, s	16	16	16

[a]Recorded at 800 MHz (^{1}H NMR) and 200 MHz (^{13}C NMR) at 25 °C.

S19: ^{1}H NMR spectrum of compound **8** in $CDCl_3$

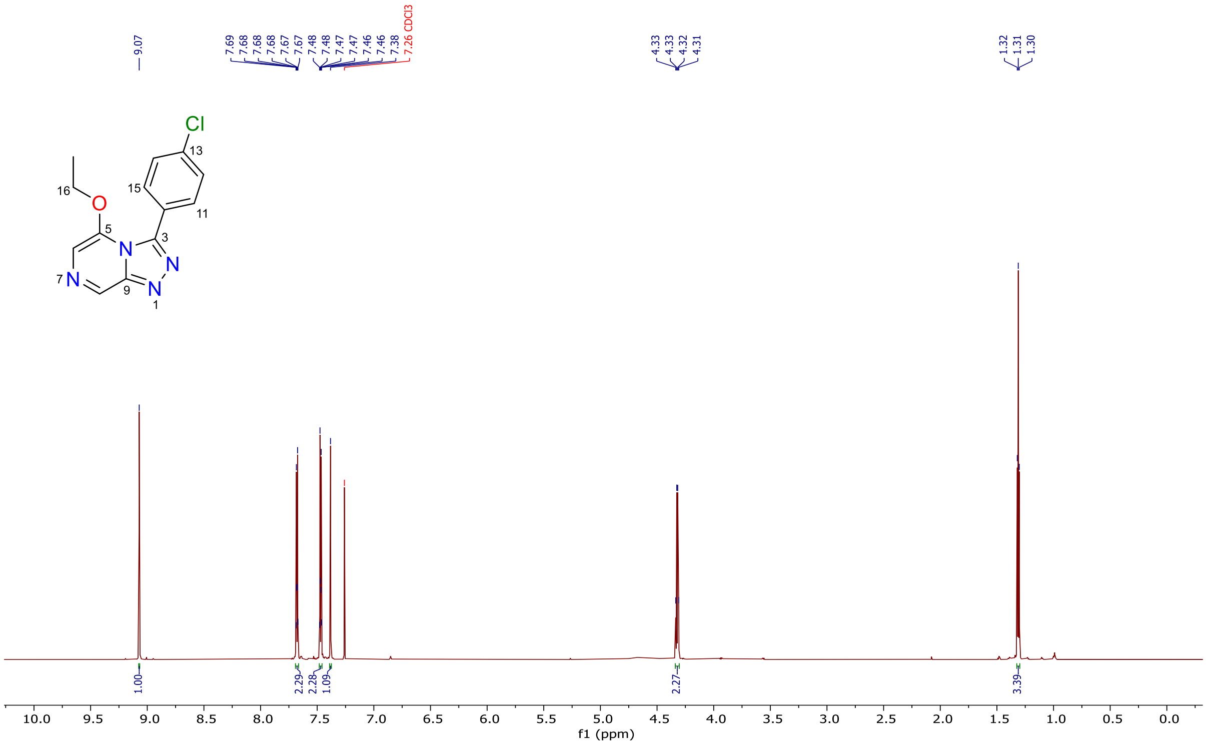

S20: ^{13}C NMR spectrum of compound **8** in $CDCl_3$

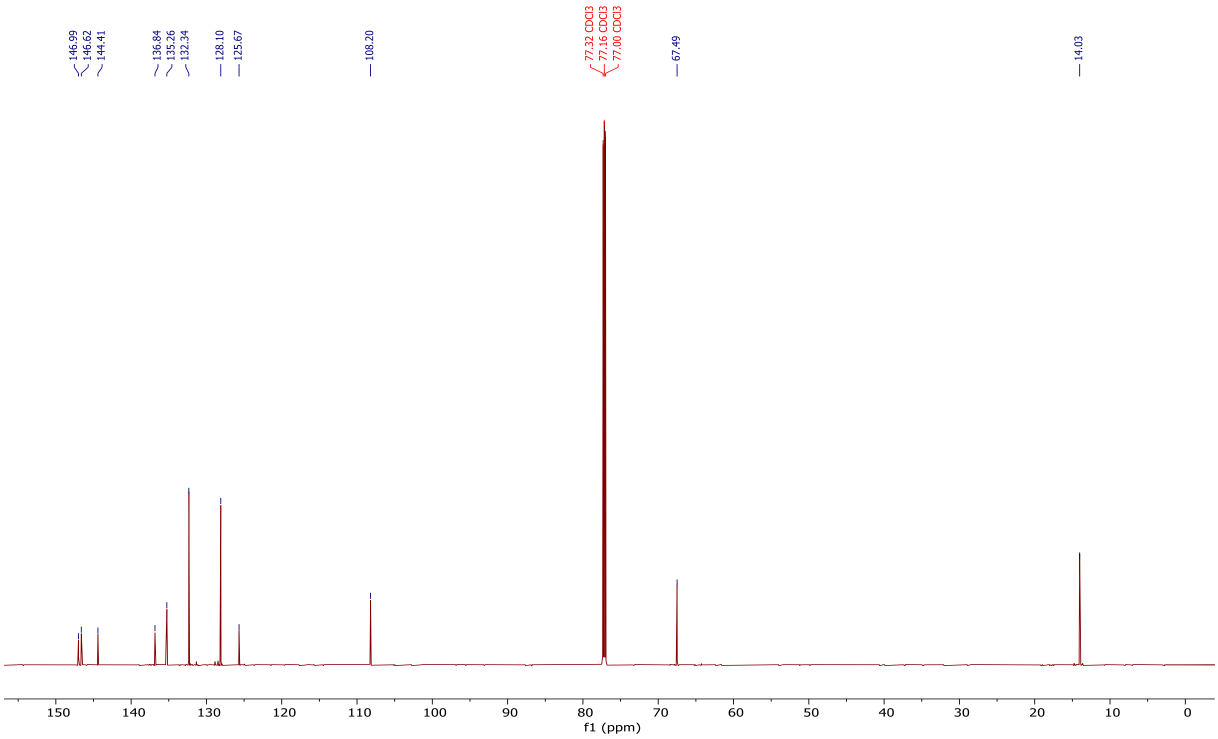

S21: *4-(5-ethoxy-[1,2,4]triazolo[4,3-a]pyrazin-3-yl)benzonitrile* (**9**)

Pale yellow amorphous solid (43 mg, 41%); UV (MeOH) λ_{max} (log ε) 223 (4.03), 246 (3.97), 297 (3.83) nm; LRESIMS *m/z* 266 $[M + H]^+$; HRESIMS *m/z* 266.1036 $[M + H]^+$ (calcd for $C_{14}H_{12}N_5O$, 266.1036).

Table S6: NMR data of compound **9** in $CDCl_3$[a]

Position	δ_H, mult. (*J* in Hz), int.	δ_C, mult. (*J* in Hz)	COSY	HMBC	ROESY
3		145.8, s			
5		144.0, s			
6	7.40, s, 1H	108.6, s		5, 8	16
8	9.08, s, 1H	135.8, s		5, 6, 9	
9		147.7, s			
10		132.0, s			
11	7.88, m, 1H	131.7, s	12	3, 13, 15	12
12	7.78, m, 1H	131.4, s	11	10, 14, 16	11
13		113.9, s			
14	7.78, m, 1H	131.4, s	15	10, 12, 16	15
15	7.88, m, 1H	131.7, s	14	3, 11, 13	14
16		118.3, s			
17	4.33, q (7.0), 2H	67.4, s	18	5, 18	6, 18
18	1.30, t (7.0), 3H	14.0, s	17	17	17

[a]Recorded at 800 MHz (^{1}H NMR) and 200 MHz (^{13}C NMR) at 25 °C.

S22: ^{1}H NMR spectrum of compound **9** in $CDCl_3$

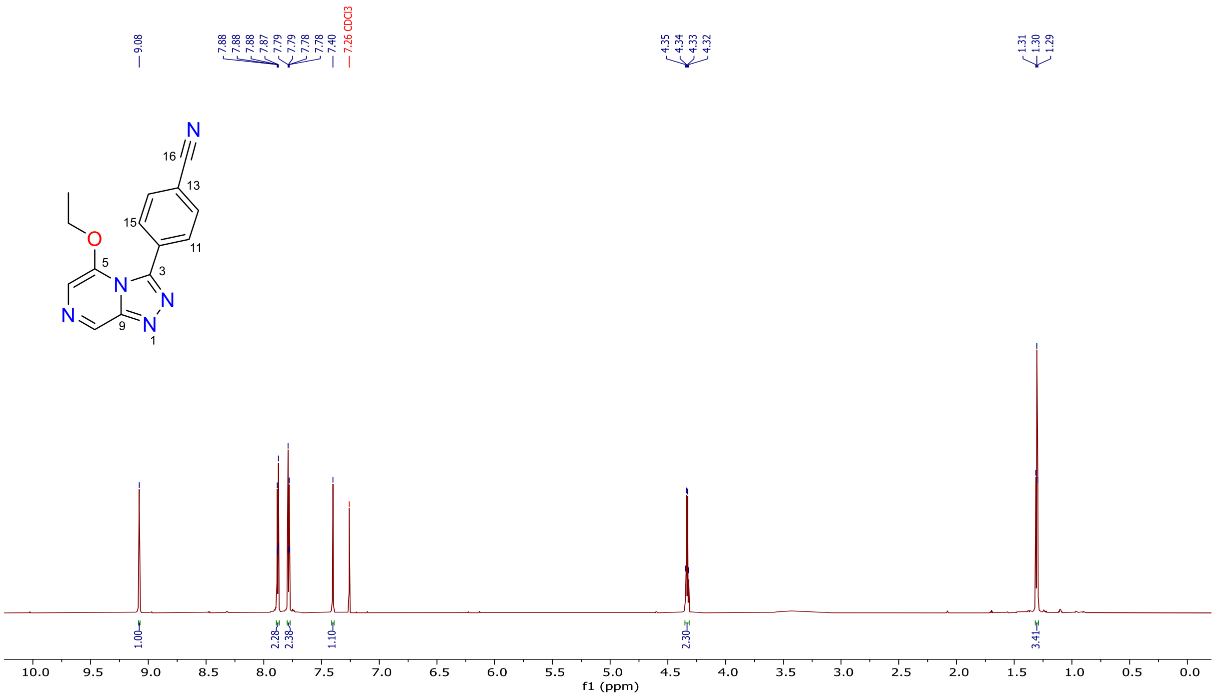

S23: ^{13}C NMR spectrum of compound **9** in $CDCl_3$

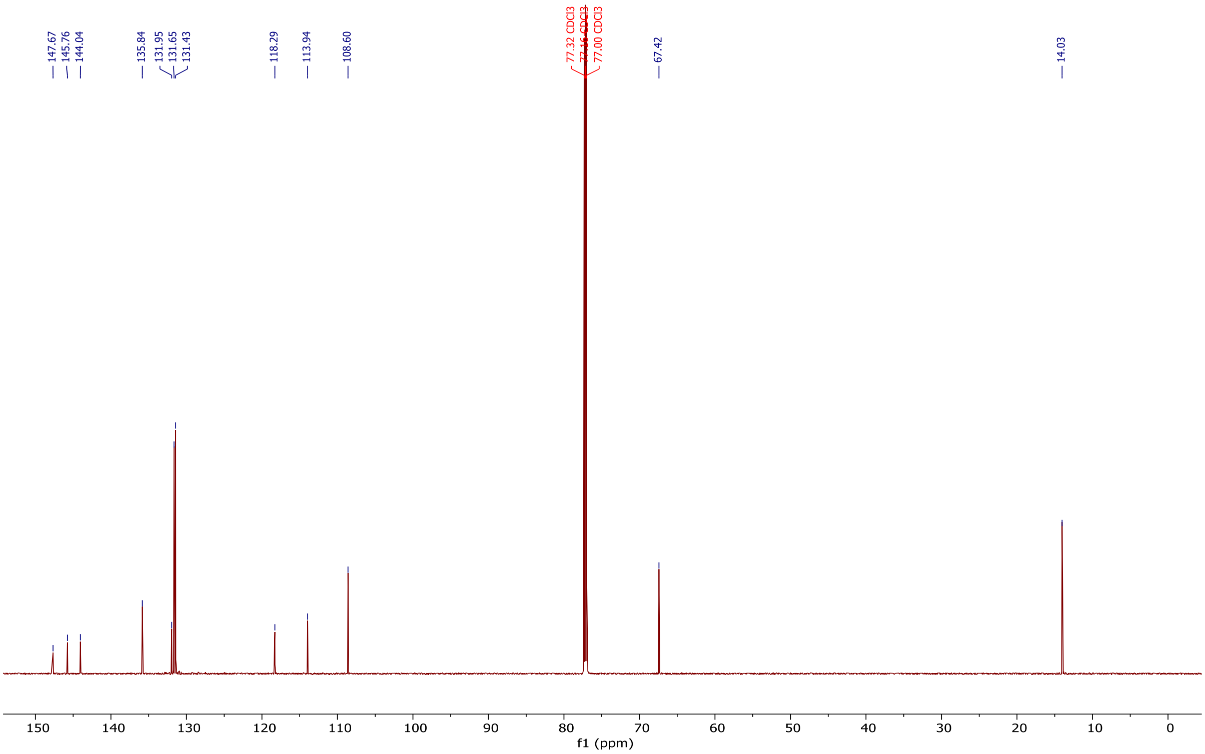

S24: NMR data of compound **10** in $CDCl_3$[a]

Position	δ_H, mult. (*J* in Hz), int.	δ_C, mult. (*J* in Hz)	COSY	HMBC	ROESY
3		147.2, s			
5		145.8, s			
6	7.37, s, 1H	106.9, s		5, 8	17
8		133.1, q (38.8)			
9		144.2, s			
10		124.2, s			
11	7.62, m, 1H	132.6, s	12	3, 13, 15	12
12	7.19, m, 1H	118.9, s	11	10, 14	11
13		152.7, t (2.7)			
14	7.19, m, 1H	118.9, s	15	10, 12	15
15	7.62, m, 1H	132.6, s	14	3, 11, 13	14
16	6.58, t (73.5), 1H	115.6, t (262.1)		13	
17	4.54, t (6.6), 2H	72.1, s	18	5, 18, 19	6, 18
18	2.96, t (6.6), 2H	34.5, s	17	17, 19, 20, 24	17, 20, 24
19		135.8, s			
20	6.86, m, 1H	128.5, s	21	18, 22, 24	18, 21
21	7.23, m, 1H	128.9, s	20	19, 23	20
22	7.22, m, 1H	127.4, s		20, 24	
23	7.23, m, 1H	128.9, s	24	19, 21	24
24	6.86, m, 1H	128.5, s	23	18, 20, 22	18, 23
25		120.2, q (273.3)			

[a]Recorded at 800 MHz (^1H NMR) and 200 MHz (^{13}C NMR) at 25 °C.

S25: ^1H NMR spectrum of compound **10** in $CDCl_3$

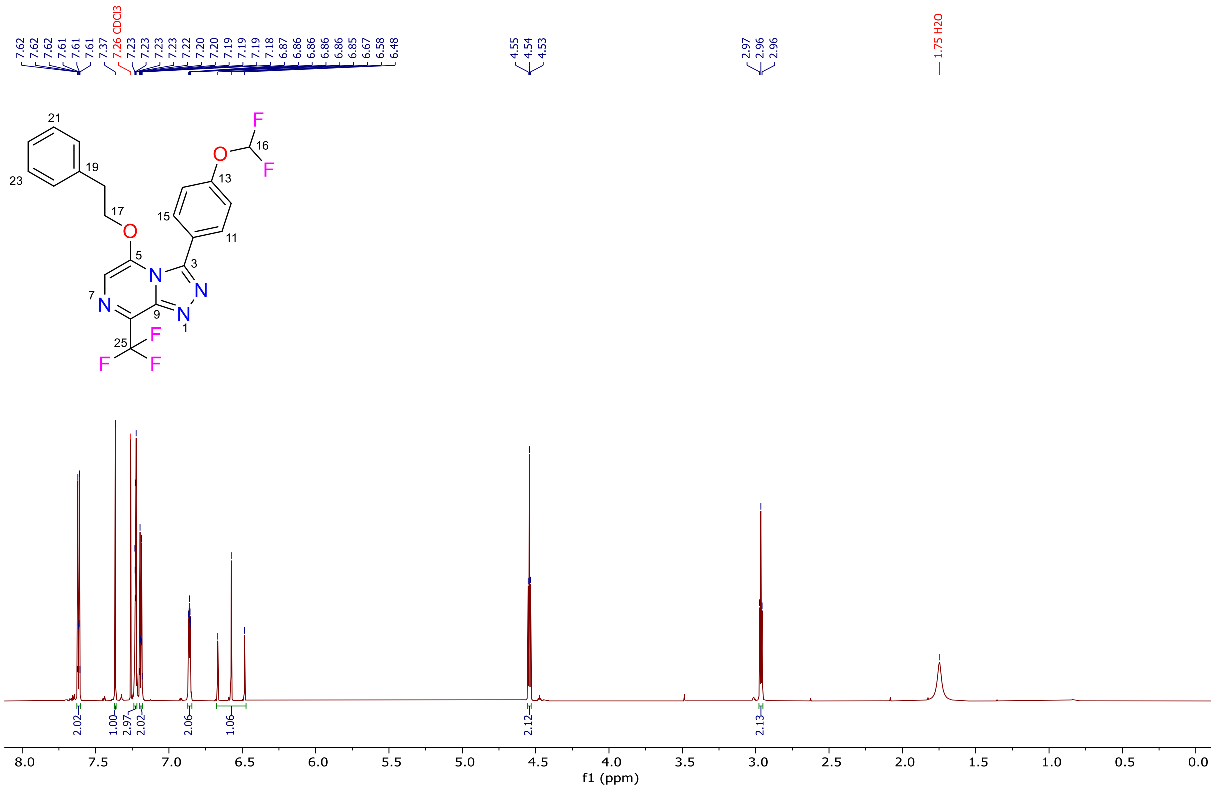

S26: ^{13}C NMR spectrum of compound **10** in $CDCl_3$

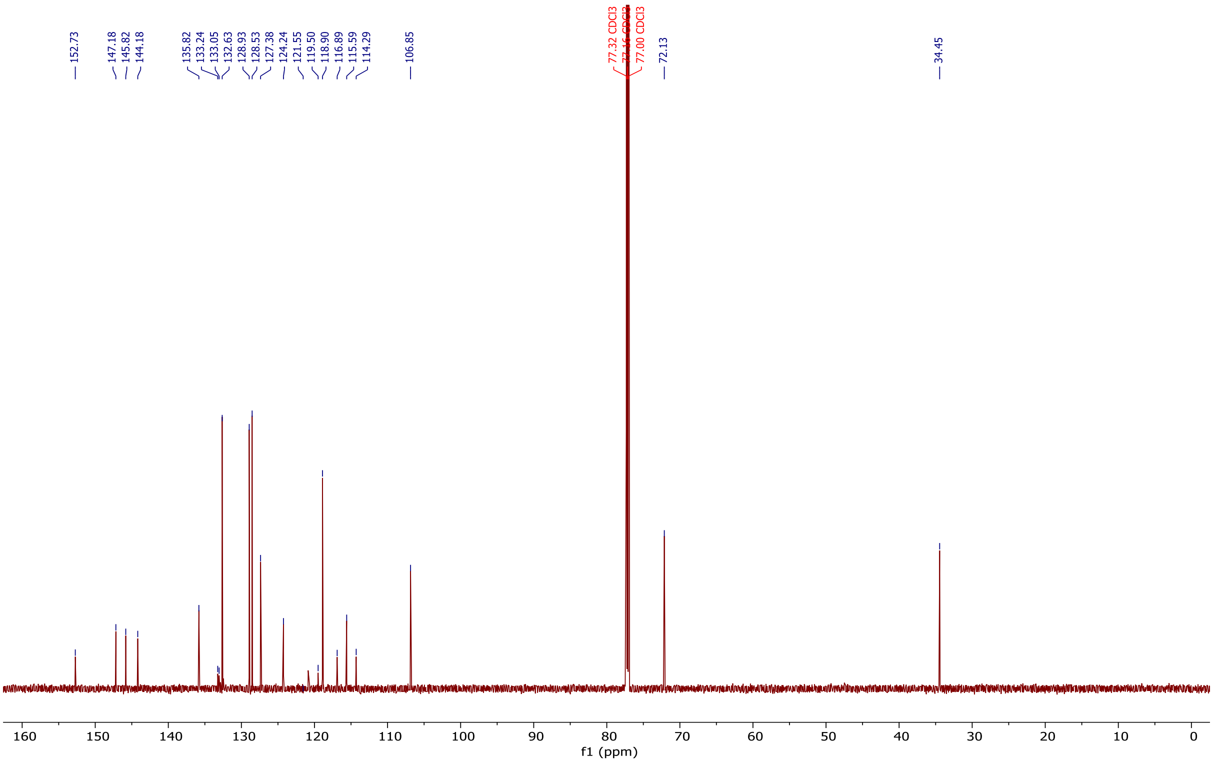

S27: NMR data of compound **11** in $CDCl_3$[a]

**Position**	**δ_H, mult. (*J* in Hz), int.**	**δ_C, mult. (*J* in Hz)**	**COSY**	**HMBC**	**ROESY**
3		146.8, s			
5		144.9, s			
6	7.30, s, 1H	106.6, s		5, 8	17
8		140.2, t (31.3)			
9		144.8, s			
10		124.7, s			
11	7.62, m, 1H	132.6, s	12	3, 13, 15	12
12	7.18, m, 1H	118.8, s	11	10, 14	11
13		152.6, t (2.8)			
14	7.18, m, 1H	118.8, s	15	10, 12	15
15	7.62, m, 1H	132.6, s	14	3, 11, 13	14
16	6.56, t (72.1), 1H	115.6, t (260.3)		13	
17	4.48, t (6.5), 2H	71.7, s	18	5, 18, 19	6, 18
18	2.94, t (6.5), 2H	34.5, s	17	19, 20, 24	17, 20, 24
19		136.1, s			
20	6.86, m, 1H	128.6, s	21	18, 22, 24	18, 21
21	7.23, m, 1H	128.9, s	20	19, 23	20
22	7.22, m, 1H	127.3, s		20, 24	
23	7.23, m, 1H	128.9, s	24	19, 21	24
24	6.86, m, 1H	128.6, s	23	18, 20, 22	18, 23
25		119.7, t (241.1)			
26	2.27, t (18.7), 3H	23.2, t (26.3)		8, 25	

[a]Recorded at 800 MHz (^{1}H NMR) and 200 MHz (^{13}C NMR) at 25 °C.

S28: ^{1}H NMR spectrum of compound **11** in $CDCl_3$

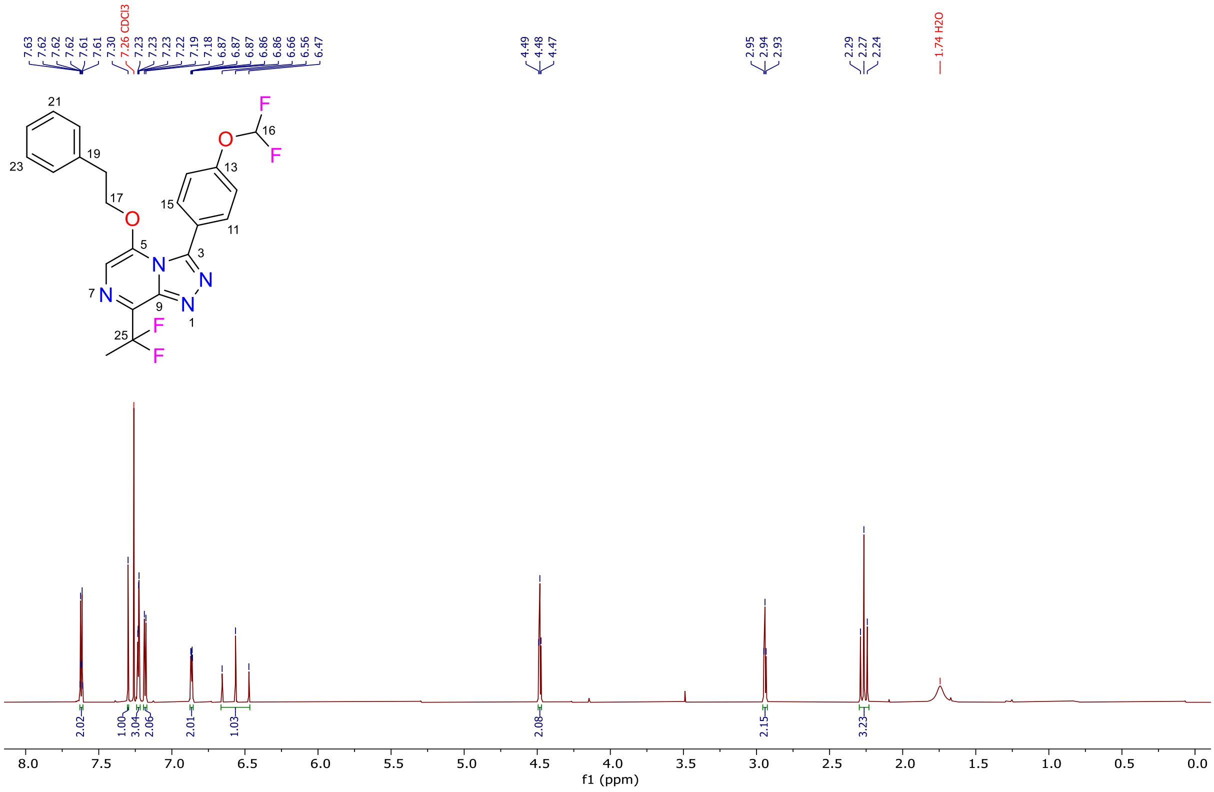

S29: ^{13}C NMR spectrum of compound **11** in $CDCl_3$

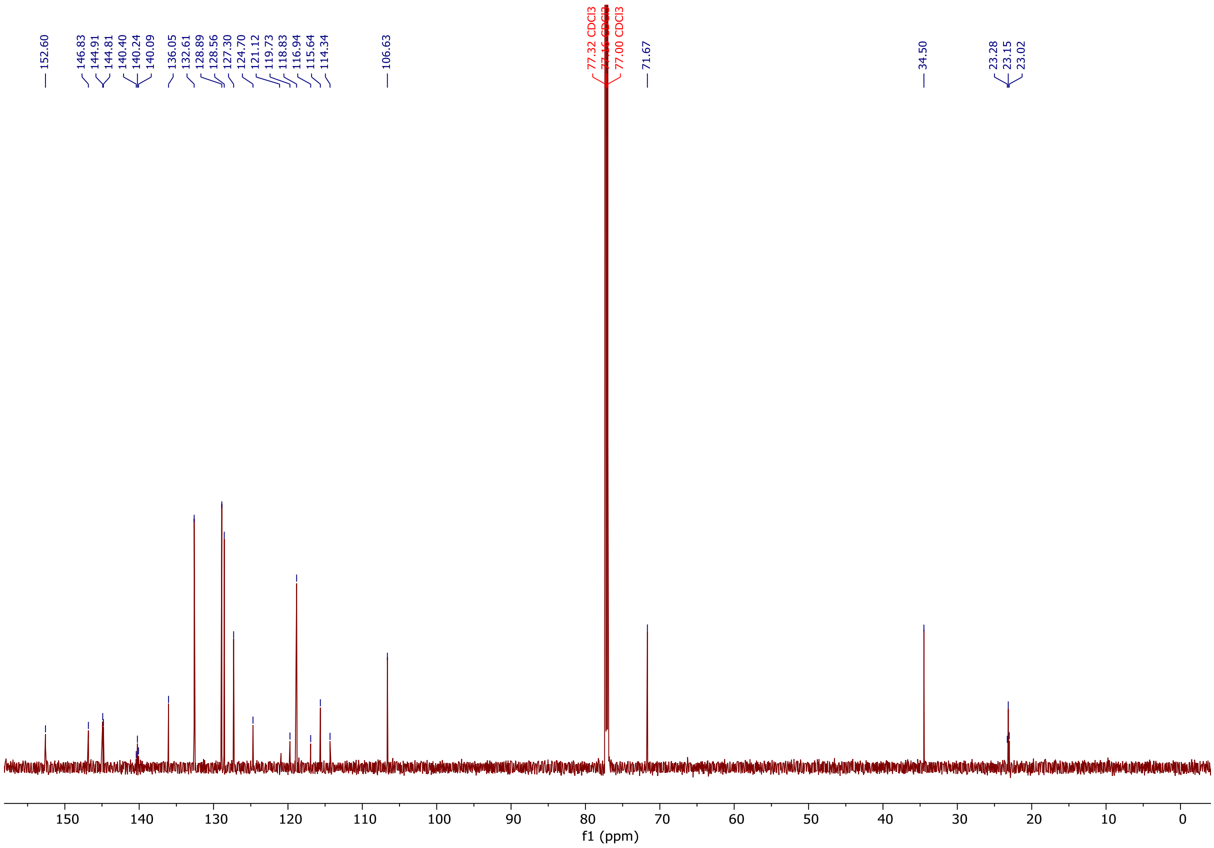

S30: NMR data of compound **12** in $CDCl_3$[a]

Position	δ_H, mult. (*J* in Hz), int.	δ_C, mult. (*J* in Hz)	COSY	HMBC	ROESY
3		147.0, s			
5		145.2, s			
6	7.34, s, 1H	107.5, s		5, 8	17
8		137.5, t (26.4)			
9		145.0, s			
10		124.4, s			
11	7.61, m, 1H	132.6, s	12	3, 13, 15	12
12	7.18, m, 1H	118.8, s	11	10, 14	11
13		152.7, t (2.7)			
14	7.18, m, 1H	118.8, s	15	10, 12	15
15	7.61, m, 1H	132.6, s	14	3, 11, 13	14
16	6.57, t (73.0), 1H	115.6, t (262.2)		13	
17	4.52, t (6.5), 2H	71.9, s	18	5, 18, 19	6, 18
18	2.96, t (6.5), 2H	34.4, s	17	17, 19, 20, 24	17, 20, 24
19		136.0, s			
20	6.86, m, 1H	128.5, s	21	18, 22, 24	18, 21
21	7.22, m, 1H	128.8, s	20	19, 23	20
22	7.21, m, 1H	127.2, s		20, 24	
23	7.22, m, 1H	128.8, s	24	19, 21	24
24	6.86, m, 1H	128.5, s	23	18, 20, 22	18, 23
25	7.09, t (54.0), 1H	111.5, t (241.3)		8, 9	

[a]Recorded at 800 MHz (^{1}H NMR) and 200 MHz (^{13}C NMR) at 25 °C.

S31: ^{1}H NMR spectrum of compound **12** in $CDCl_3$

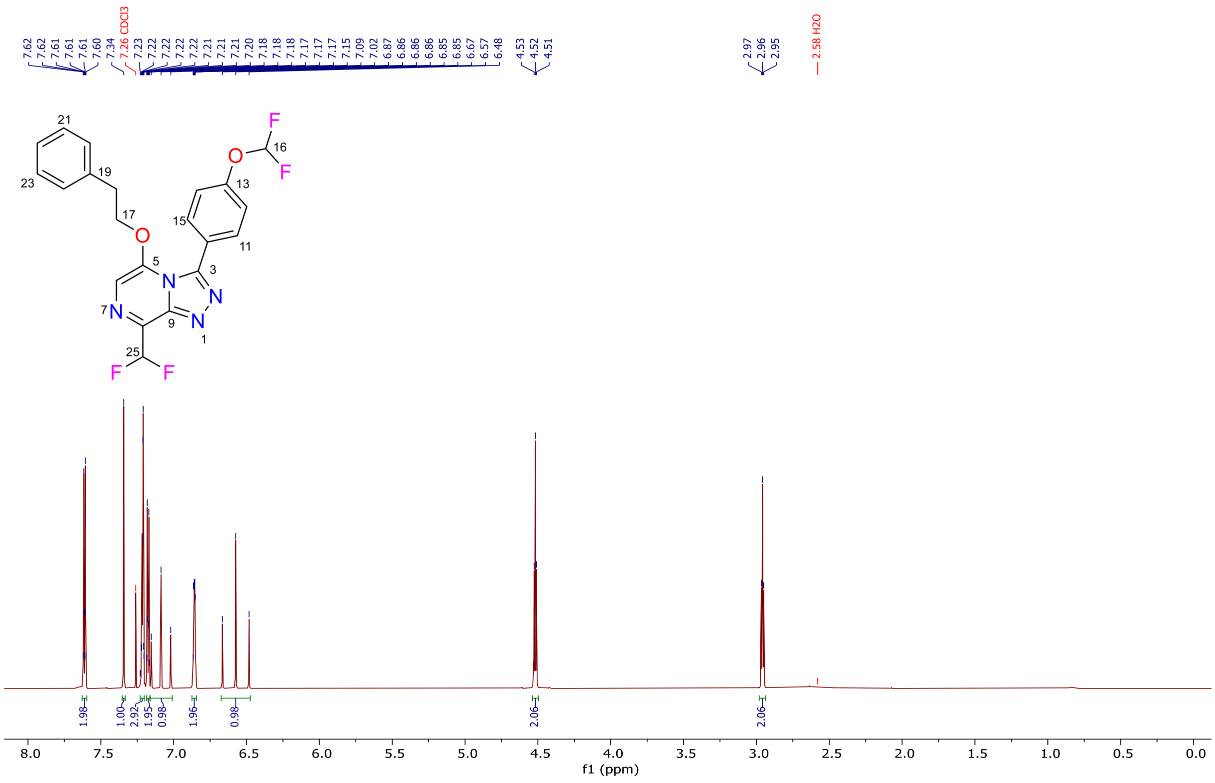

S32: ^{13}C NMR spectrum of compound **12** in $CDCl_3$

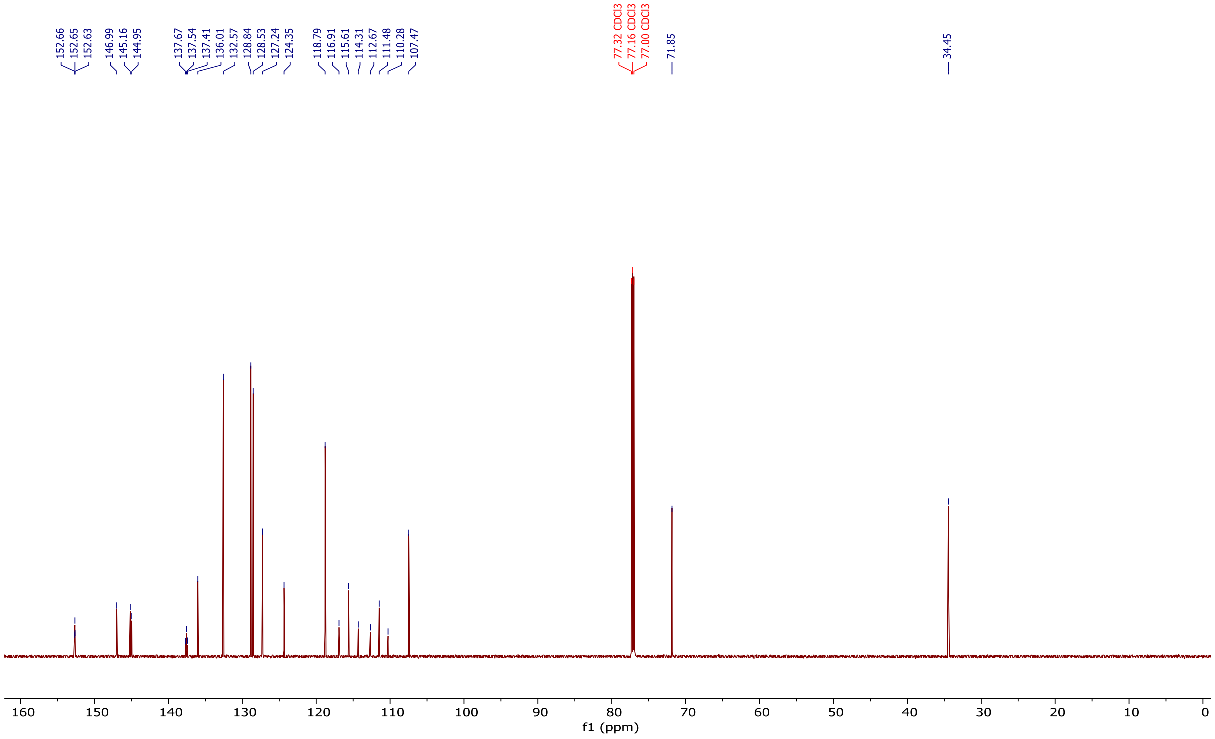

S33: NMR data of compound **13** in $CDCl_3$[a]

Position	**δ_H, mult. (*J* in Hz), int.**	**δ_C, mult. (*J* in Hz)**	**COSY**	**HMBC**	**ROESY**
3		147.2, s			
5		145.8, s			
6	7.39, s, 1H	107.0, s		5, 8	16
8		133.2, q (38.9)			
9		144.1, s			
10		125.4, s			
11	7.54, m, 1H	132.2, s	12	3, 13, 15	12
12	7.42, m, 1H	128.3, s	11	10, 14	11
13		137.1, s			
14	7.42, m, 1H	128.3, s	15	10, 12	15
15	7.54, m, 1H	132.2, s	14	3, 11, 13	14
16	4.55, t (6.6), 2H	72.2, s	17	5, 17, 18	6, 17
17	2.98, t (6.6), 2H	34.5, s	16	16, 18, 19, 23	16, 19, 23
18		135.7, s			
19	6.88, m, 1H	128.5, s	20	17, 21, 23	17, 20
20	7.25, m, 1H	129.0, s	19	18, 22	19
21	7.24, m, 1H	127.4, s		19, 23	
22	7.25, m, 1H	129.0, s	23	20, 20	23
23	6.88, m, 1H	128.5, s	22	17, 19, 21	17, 22
24		120.0, q (274.4)			

[a]Recorded at 800 MHz (^{1}H NMR) and 200 MHz (^{13}C NMR) at 25 °C.

S34: ^{1}H NMR spectrum of compound **13** in $CDCl_3$

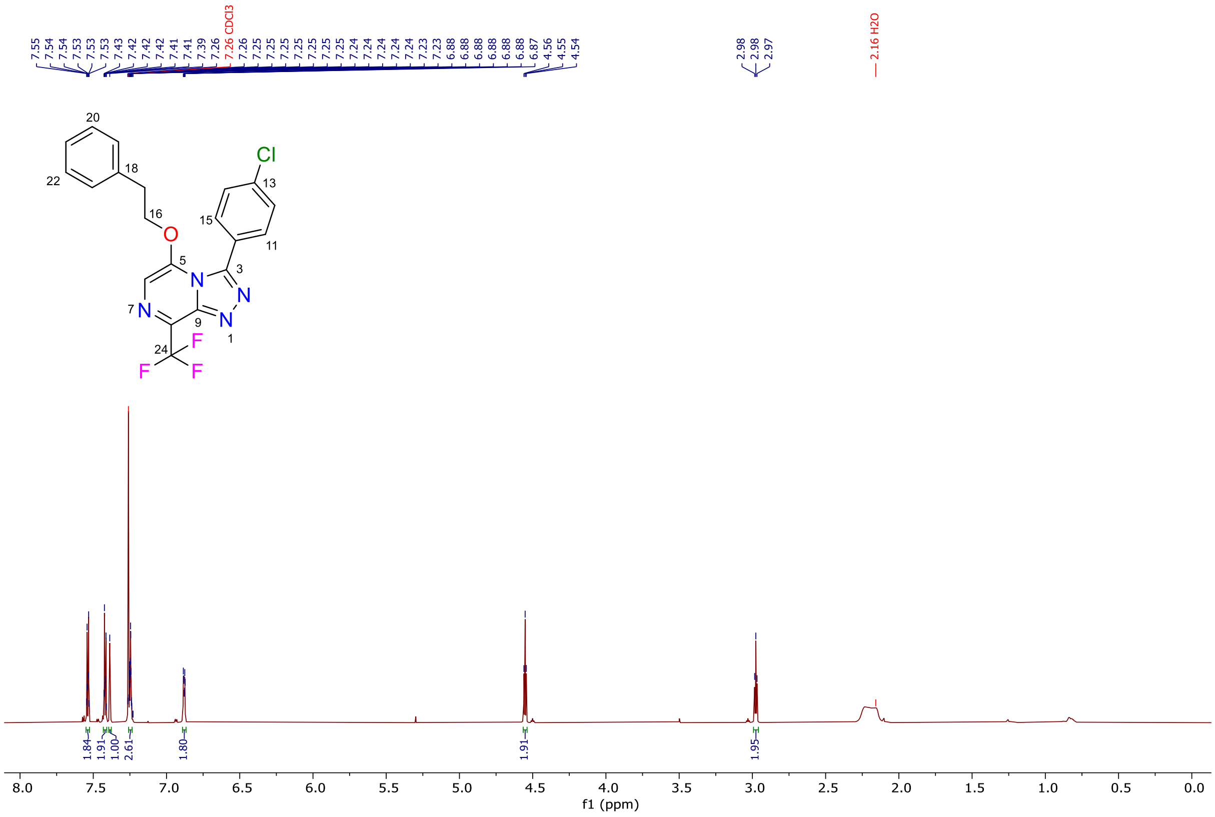

S35: ^{13}C NMR spectrum of compound **13** in $CDCl_3$

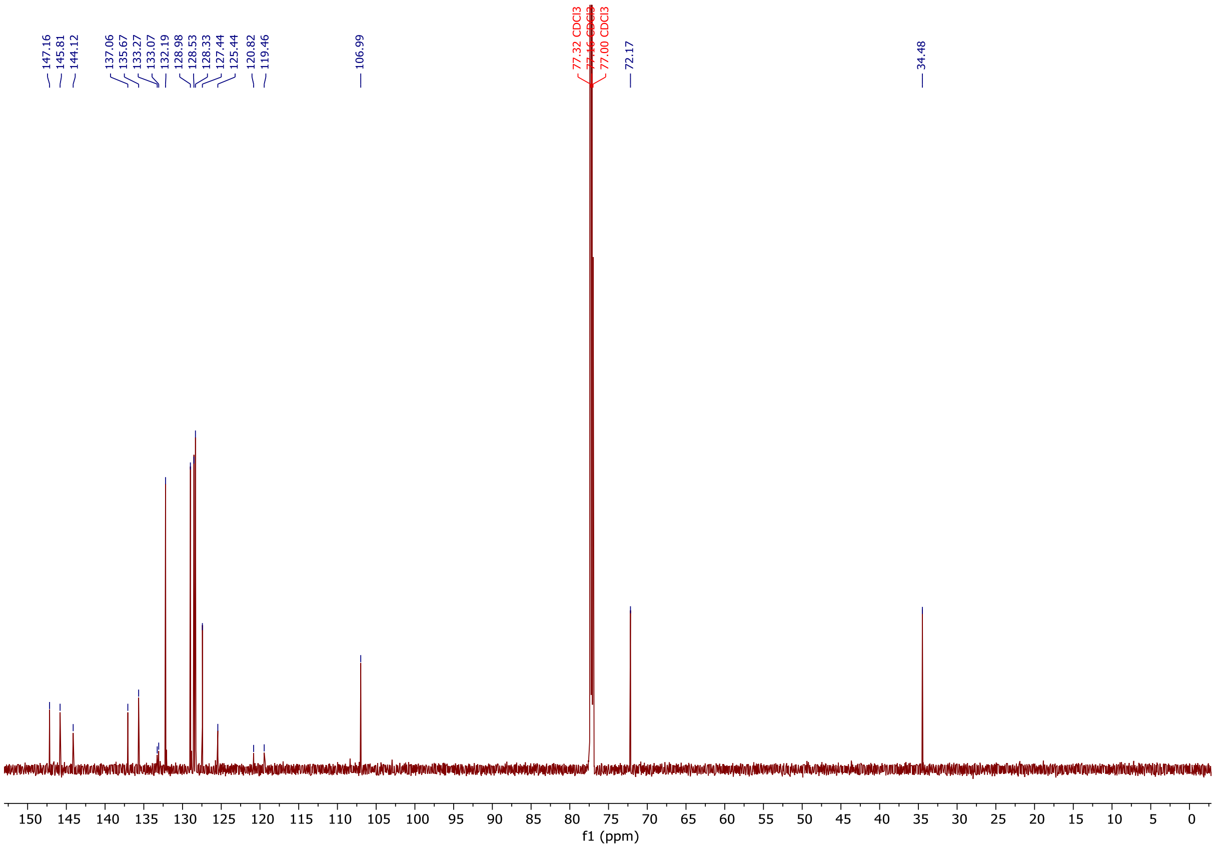

S36: NMR data of compound **14** in $CDCl_3$[a]

Position	δ_H, mult. (J in Hz), int.	δ_C, mult. (J in Hz)	COSY	HMBC	ROESY
3		146.8, s			
5		144.8, s			
6	7.30, s, 1H	106.7, s		5, 8	16
8		140.2, t (31.7)			
9		144.9, s			
10		126.0, s			
11	7.53, m, 1H	132.2, s	12	3, 13, 15	12
12	7.40, m, 1H	128.2, s	11	10, 14	11
13		136.7, s			
14	7.40, m, 1H	128.2, s	15	10, 12	15
15	7.53, m, 1H	132.2, s	14	3, 11, 13	14
16	4.49, t (6.5), 2H	71.7, s	17	5, 17, 18	6, 17
17	2.95, t (6.5), 2H	34.5, s	16	16, 18, 19, 23	16, 19, 23
18		136.0, s			
19	6.88, m, 1H	128.6, s	20	17, 21, 23	17, 20
20	7.24, m, 1H	128.9, s	19	18, 22	19
21	7.23, m, 1H	127.3, s		19, 23	
22	7.24, m, 1H	128.9, s	23	20, 20	23
23	6.88, m, 1H	128.6, s	22	17, 19, 21	17, 22
24		119.7, t (240.3)			
25	2.26, t (18.9), 3H	23.1, t (26.2)		8, 24	

[a]Recorded at 800 MHz (^1H NMR) and 200 MHz (^{13}C NMR) at 25 °C.

S37: ^1H NMR spectrum of compound **14** in $CDCl_3$

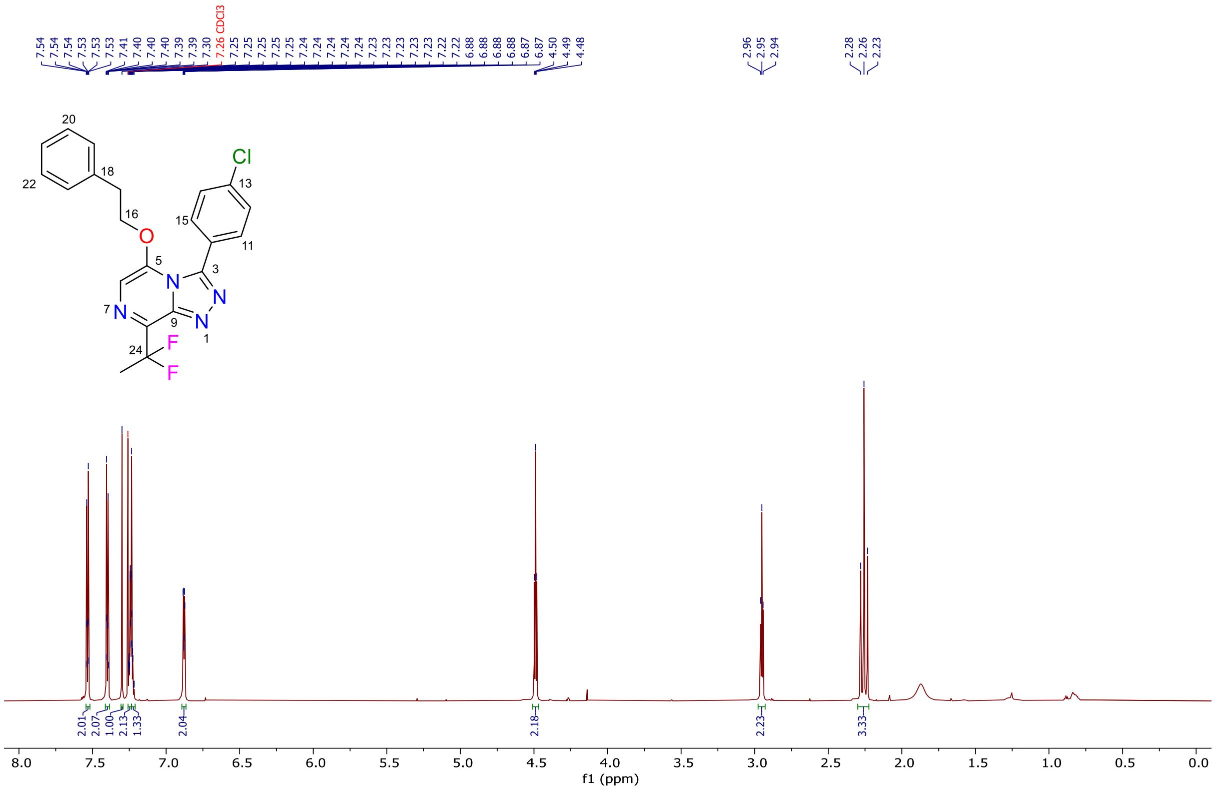

S38: ^{13}C NMR spectrum of compound **14** in $CDCl_3$

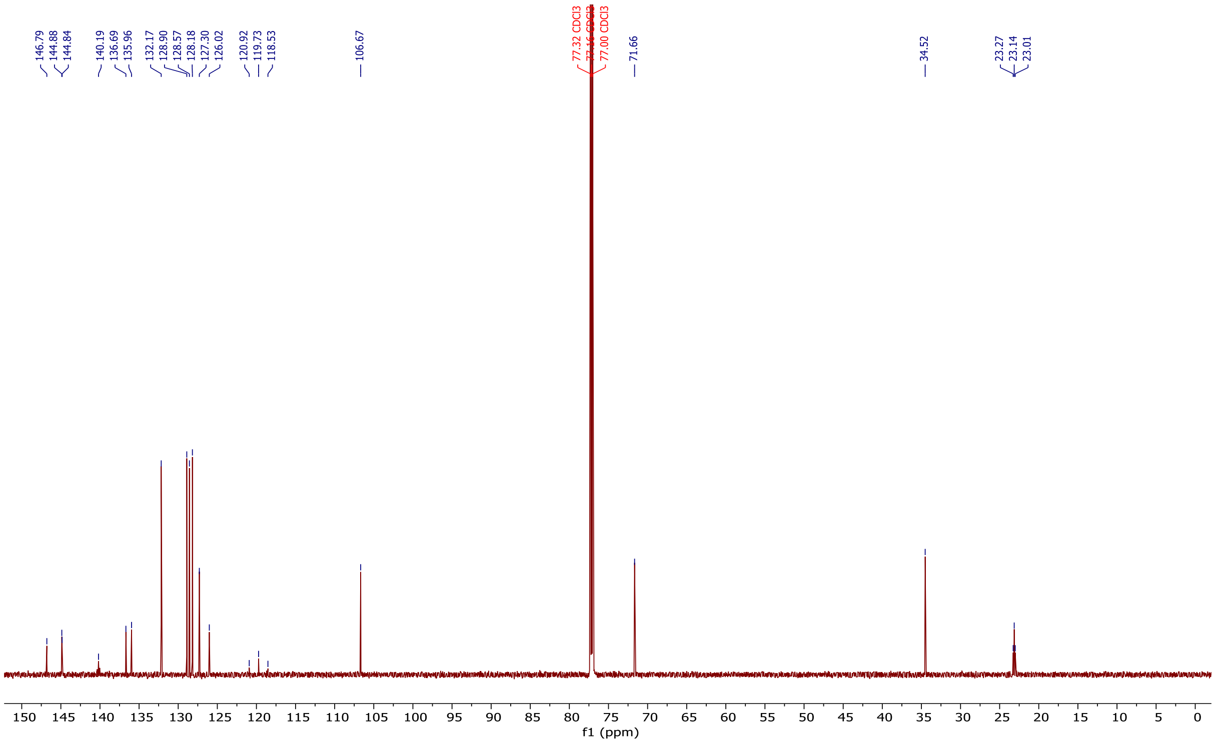

S39: NMR data of compound **15** in $CDCl_3$[a]

Position	δ_H, mult. (*J* in Hz), int.	δ_C, mult. (*J* in Hz)	COSY	HMBC	ROESY
3		147.0, s			
5		145.2, s			
6	7.40, s, 1H	107.8, s		5, 8	16
8		137.3, t (26.6)			
9		144.5, s			
10		125.1, s			
11	7.52, m, 1H	132.1, s	12	3, 13, 15	12
12	7.40, m, 1H	128.3, s	11	10, 14	11
13		137.1, s			
14	7.40, m, 1H	128.3, s	15	10, 12	15
15	7.52, m, 1H	132.1, s	14	3, 11, 13	14
16	4.54, t (6.5), 2H	72.1, s	17	5, 17, 18	6, 17
17	2.97, t (6.5), 2H	34.4, s	16	16, 18, 19, 23	16, 19, 23
18		135.8, s			
19	6.87, m, 1H	128.5, s	20	17, 21, 23	17, 20
20	7.23, m, 1H	128.9, s	19	18, 22	19
21	7.22, m, 1H	127.3, s		19, 23	
22	7.23, m, 1H	128.9, s	23	20, 20	23
23	6.87, m, 1H	128.5, s	22	17, 19, 21	17, 22
24	7.05, t (53.5), 1H	111.6, t (241.8)		8, 9	

[a]Recorded at 800 MHz (^{1}H NMR) and 200 MHz (^{13}C NMR) at 25 °C.

S40: ^{1}H NMR spectrum of compound **15** in $CDCl_3$

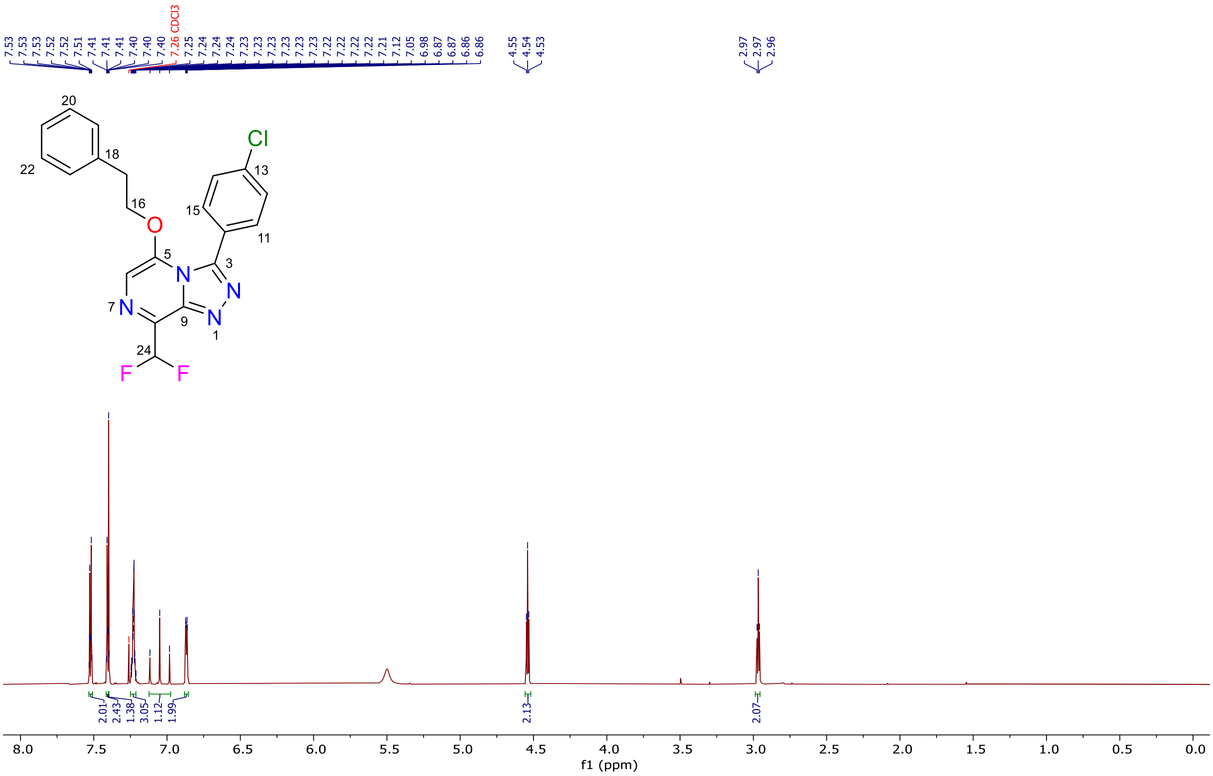

S41: ^{13}C NMR spectrum of compound **15** in $CDCl_3$

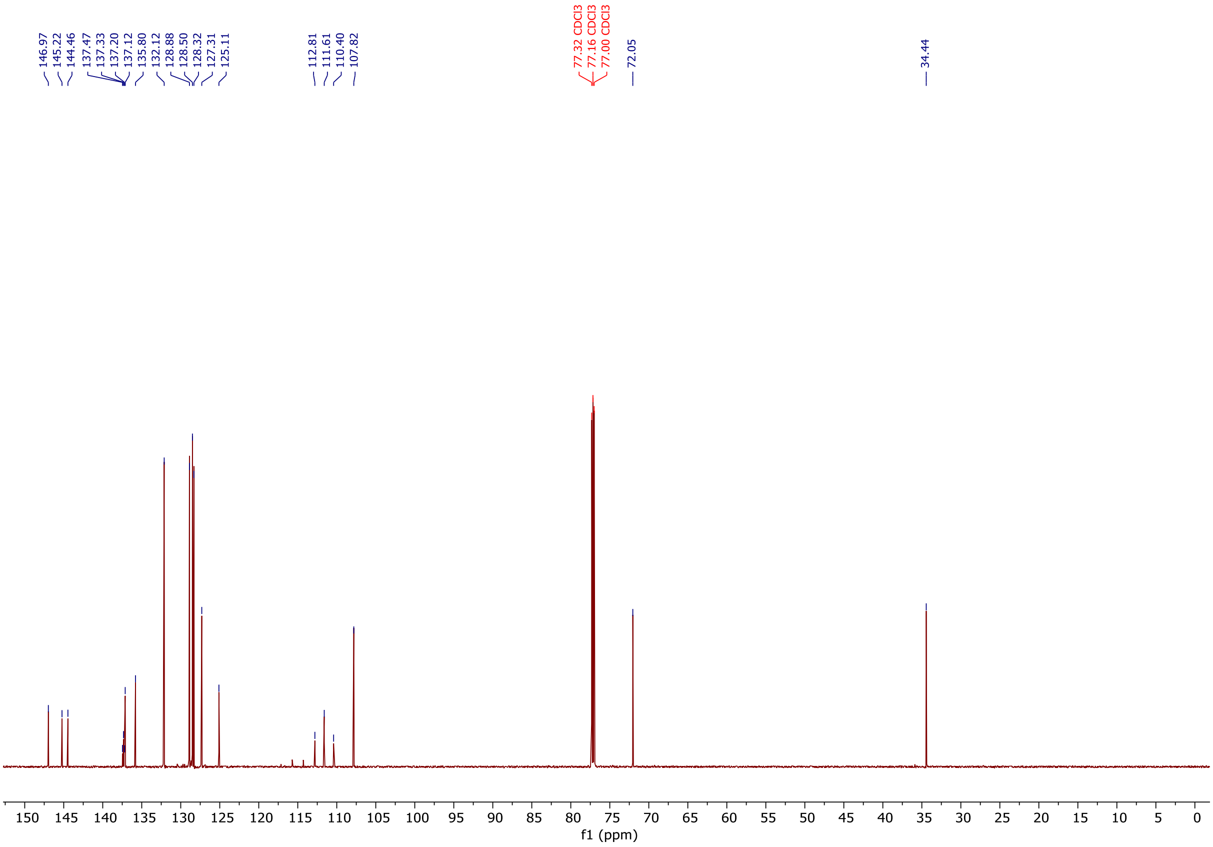

S42: NMR data of compound **16** in $CDCl_3$[a]

Position	δ_H, mult. (*J* in Hz), int.	δ_C, mult. (*J* in Hz)	COSY	HMBC	ROESY
3		146.4, s			
5		145.6, s			
6	7.47, s, 1H	107.6, s		5, 8	17, 18
8		133.1, q (39.1)			
9		144.1, s			
10		131.1, s			
11	7.68, m, 1H	131.5, s	12	3, 13, 15	12
12	7.64, m, 1H	131.6, s	11	10, 14, 16	11
13		114.3, s			
14	7.64, m, 1H	131.6, s	15	10, 12, 16	15
15	7.68, m, 1H	131.5, s	14	3, 11, 13	14
16		118.1, s			
17	4.62, t (6.5), 2H	72.1, s	18	5, 18, 19	6, 18, 20, 24
18	2.99, t (6.5), 2H	34.2, s	17	17, 19, 20, 24	6, 17, 20, 24
19		135.4, s			
20	6.90, m, 1H	128.3, s	21	18, 22, 24	17, 18, 21
21	7.27, m, 1H	129.1, s	20	19, 23	20
22	7.26, m, 1H	127.6, s		20, 24	
23	7.27, m, 1H	129.1, s	24	19, 21	24
24	6.90, m, 1H	128.3, s	23	18, 20, 22	17, 18, 23
25		120.0, q (274.6)			

[a]Recorded at 800 MHz (^{1}H NMR) and 200 MHz (^{13}C NMR) at 25 °C.

S43: ^{1}H NMR spectrum of compound **16** in $CDCl_3$

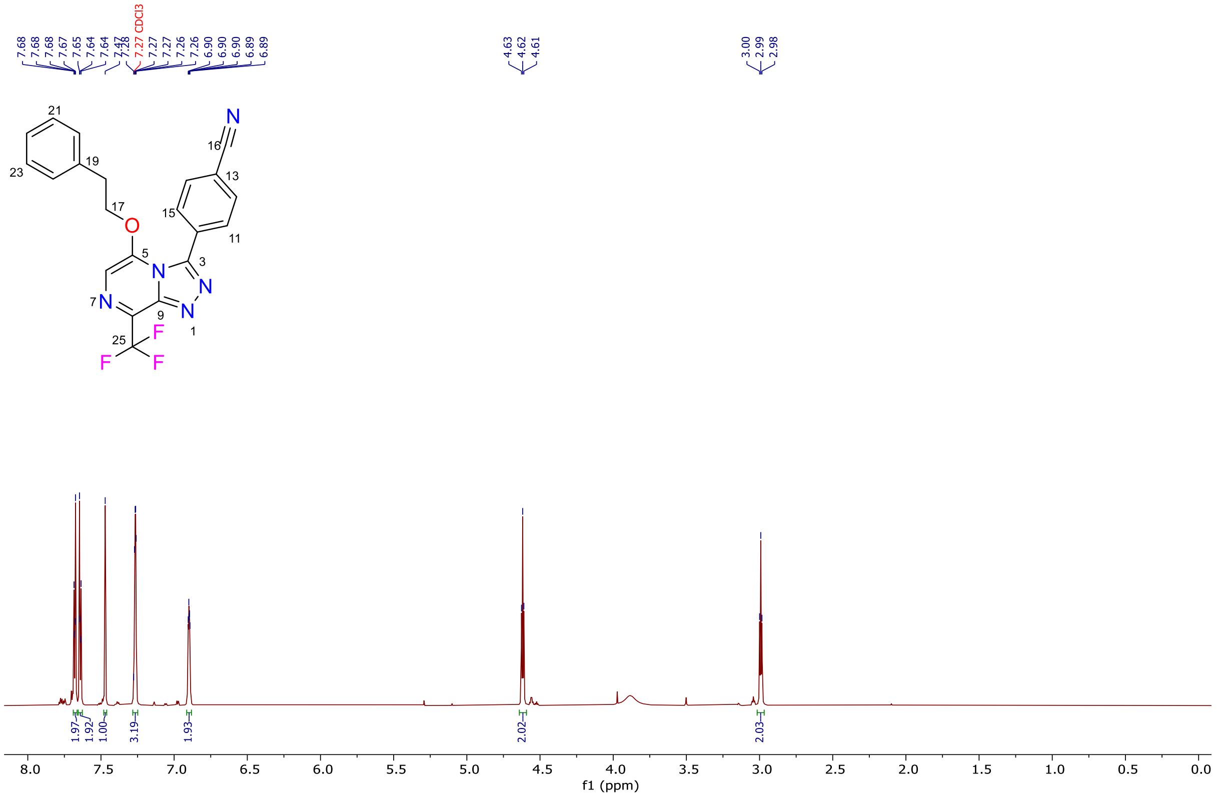

S44: ^{13}C NMR spectrum of compound **16** in $CDCl_3$

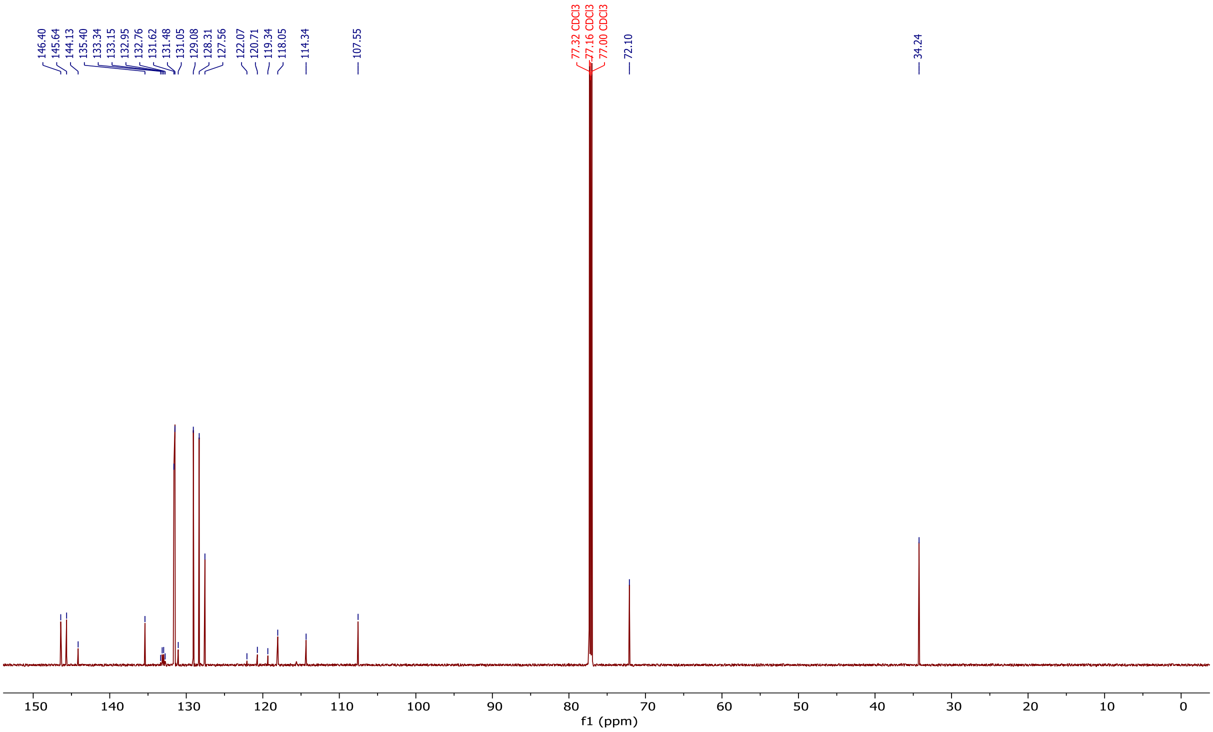

S45: NMR data of compound **17** in $CDCl_3$[a]

Position	δ_H, mult. (*J* in Hz), int.	δ_C, mult. (*J* in Hz)	COSY	HMBC	ROESY
3		146.1, s			
5		144.7, s			
6	7.38, s, 1H	107.0, s		5, 8	17
8		140.4, t (31.7)			
9		145.0, s			
10		131.9, s			
11	7.69, m, 1H	131.51, s	12	3, 13, 15	12
12	7.64, m, 1H	131.46, s	11	10, 14, 16	11
13		114.0, s			
14	7.64, m, 1H	131.46, s	15	10, 12, 16	15
15	7.69, m, 1H	131.51, s	14	3, 11, 13	14
16		118.3, s			
17	4.55, t (6.5), 2H	71.5, s	18	5, 18, 19	6, 18, 20, 24
18	2.97, t (6.5), 2H	34.3, s	17	19, 20, 24	17, 20, 24
19		135.7, s			
20	6.90, m, 1H	128.4, s	21	18, 22, 24	17, 18, 21
21	7.27, m, 1H	129.0, s	20	19, 23	20
22	7.27, m, 1H	127.5, s		20, 24	
23	7.27, m, 1H	129.0, s	24	19, 21	24
24	6.90, m, 1H	128.4, s	23	18, 20, 22	17, 18, 23
25		119.7, t (240.3)			
26	2.27, t (18.7), 3H	23.1, t (26.4)		8, 25	

[a]Recorded at 800 MHz (^{1}H NMR) and 200 MHz (^{13}C NMR) at 25 °C.

S46: ^{1}H NMR spectrum of compound **17** in $CDCl_3$

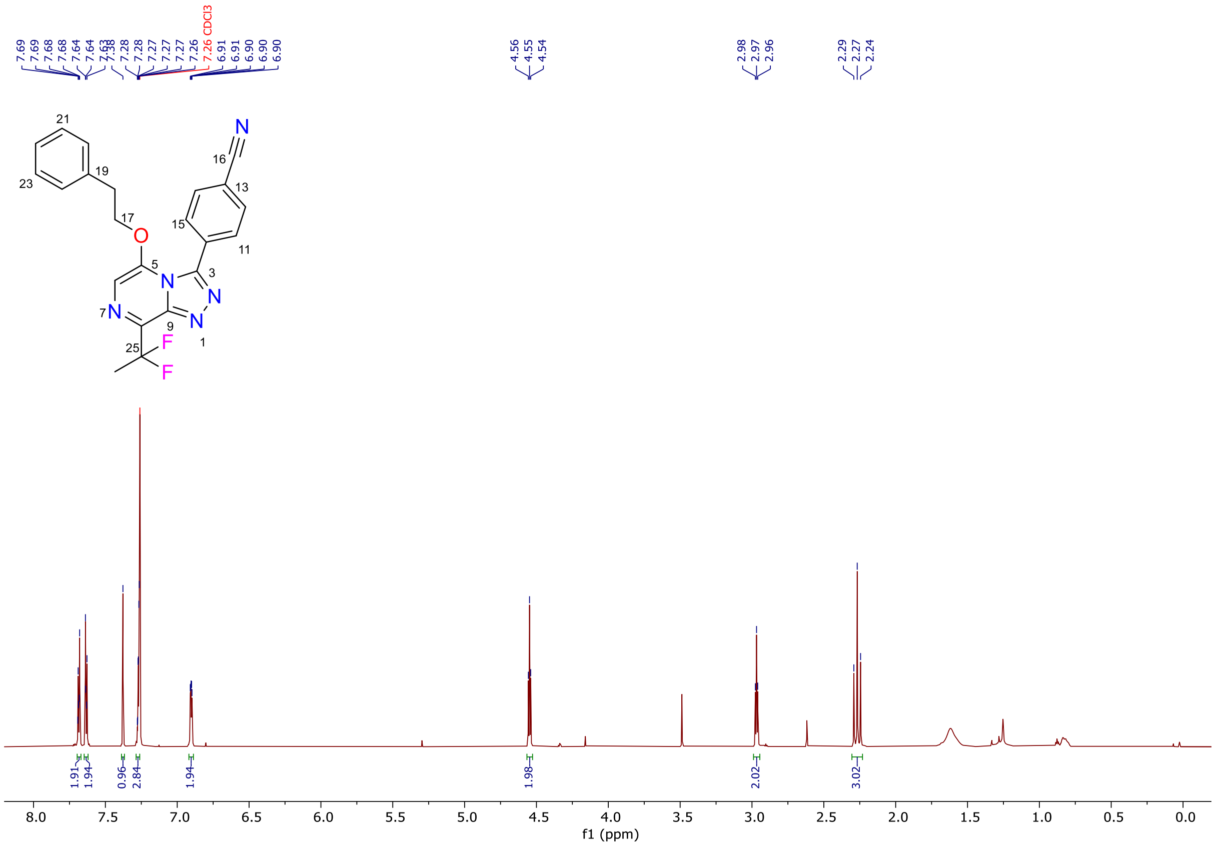

S47: ^{13}C NMR spectrum of compound **17** in $CDCl_3$

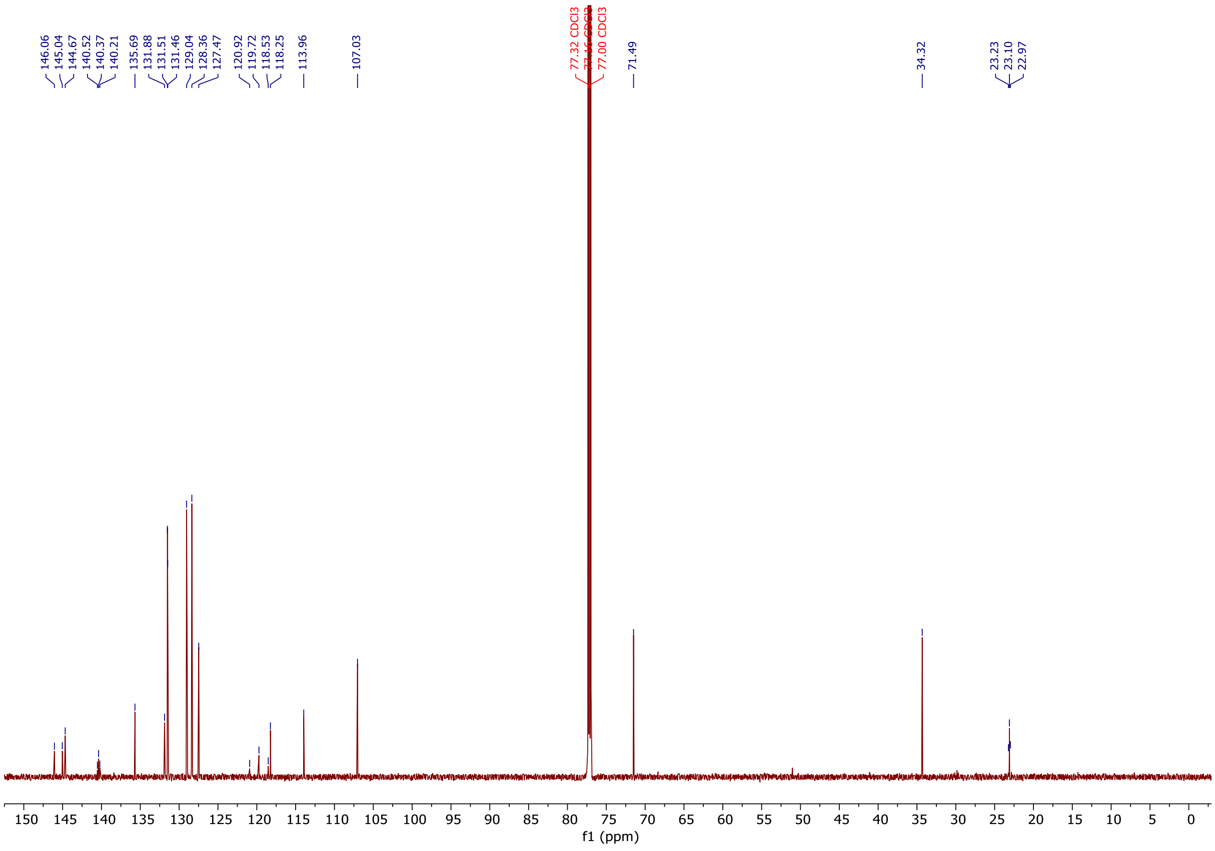

S48: NMR data of compound **18** in $CDCl_3$[a]

Position	δ_H, mult. (*J* in Hz), int.	δ_C, mult. (*J* in Hz)	COSY	HMBC	ROESY
3		146.2, s			
5		145.0, s			
6	7.49, s, 1H	108.2, s		5, 8	17
8		137.5, t (27.3)			
9		144.6, s			
10		131.0, s			
11	7.68, m, 1H	131.5, s	12	3, 13, 15	12
12	7.64, m, 1H	131.6, s	11	10, 14, 16	11
13		114.3, s			
14	7.64, m, 1H	131.6, s	15	10, 12, 16	15
15	7.68, m, 1H	131.5, s	14	3, 11, 13	14
16		118.0, s			
17	4.60, t (6.5), 2H	71.9, s	18	5, 18, 19	6, 18, 20, 24
18	2.99, t (6.5), 2H	34.3, s	17	19, 20, 24	17, 20, 24
19		135.5, s			
20	6.90, m, 1H	128.3, s	21	18, 22, 24	17, 18, 21
21	7.26, m, 1H	129.0, s	20	19, 23	20
22	7.26, m, 1H	127.5, s		20, 24	
23	7.26, m, 1H	129.0, s	24	19, 21	24
24	6.90, m, 1H	128.3, s	23	18, 20, 22	17, 18, 23
25	7.06, t (53.4), 1H	111.6, t (241.7)		8, 9	

[a]Recorded at 800 MHz (^1H NMR) and 200 MHz (^{13}C NMR) at 25 °C.

S49: ^1H NMR spectrum of compound **18** in $CDCl_3$

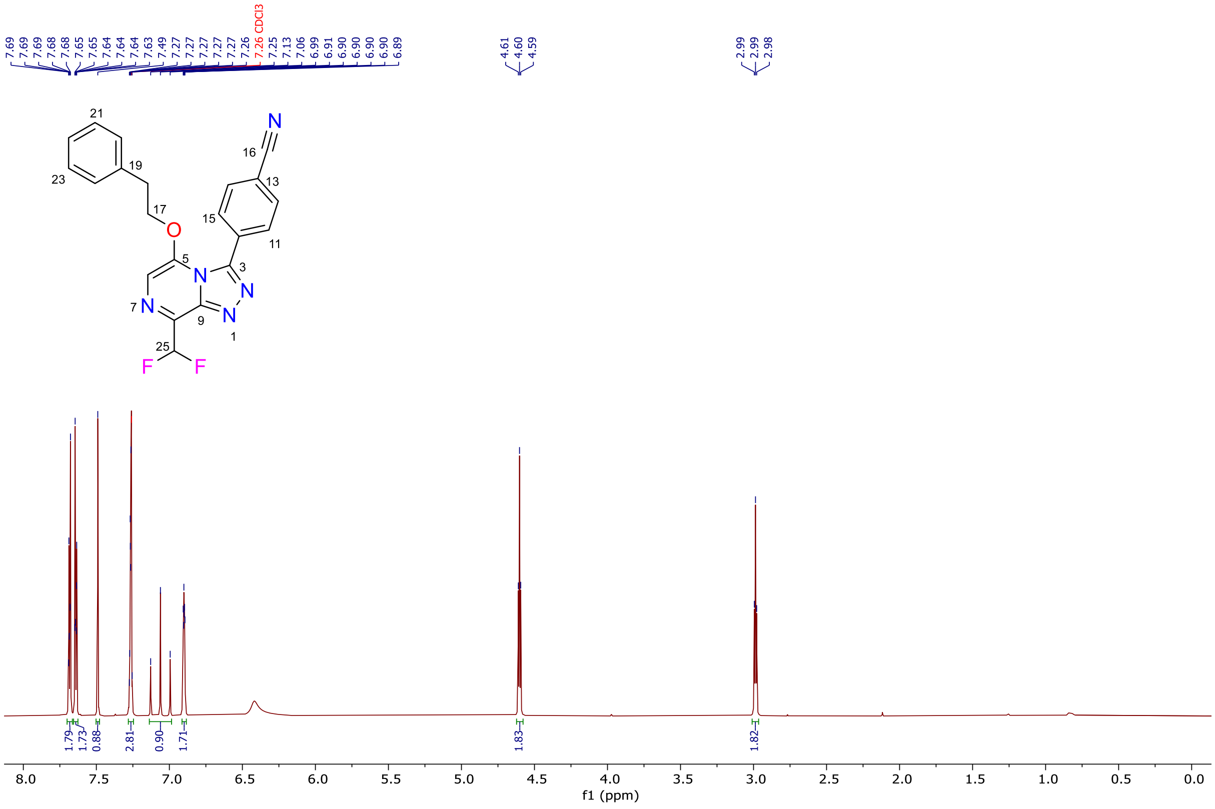

S50: ^{13}C NMR spectrum of compound **18** in $CDCl_3$

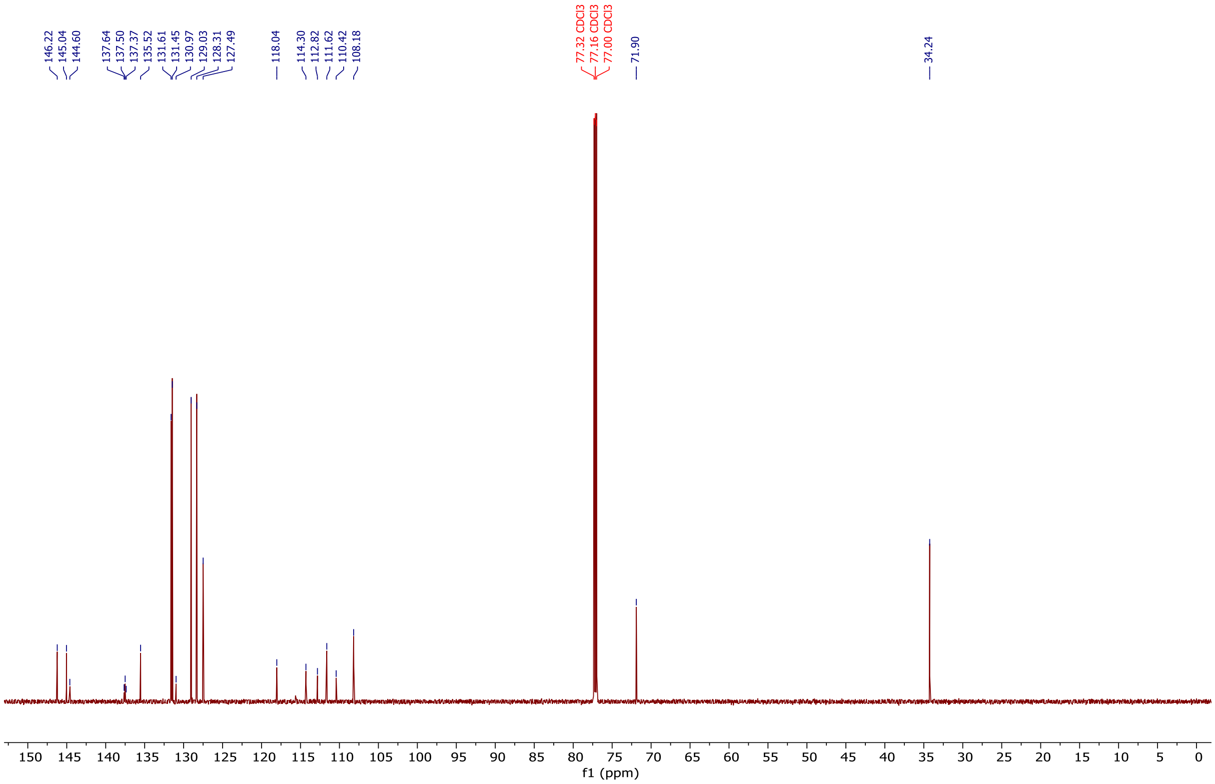

S51: Crystal data for compound **5**

$C_{19}H_{15}ClN_4O$, M = 350.80, T = 100.0(10) K, λ = 0.71073 Å, monoclinic, space group I2/a, a = 19.5831(3) Å, b = 8.89750(10) Å, c = 18.6547(3) Å, β= 93.5860(10)° V = 3244.04(8) Å^3, Z = 8, D_c = 1.437 Mg M^3 μ = 0.251 mm^{-1}, F(000) = 1456, crystal size 0.43 × 0.40 × 0.30 mm; θ_{max} = 51.26°, 51318 reflections measured, 17443 independent reflections (R_{int} = 0.0445); final R = 0.0439 [I > 2σ(I), 12147 data] and wR(F_2) = 0.1395 (all data); GOOF =1.045.

S52: Crystal data for compound **6**

$C_{20}H_{15}N_5O$, M = 341.37, T = 100.0(10) K, λ = 0.71073 Å, monoclinic, space group P21/n, a = 7.6152(1) Å, b = 16.8201(3) Å, c = 13.5669(2) Å, β= 90.877(2)°, V = 1737.56(5) Å^3, Z = 4, D_c = 1.305 Mg M^3 μ = 0.085 mm^{-1}, F(000) = 712, crystal size 0.56 × 0.33 × 0.30 mm; θ_{max} = 41.09°, 95562 reflections measured, 11148 independent reflections (R_{int} = 0.0417); final R = 0.0386 [I > 2σ(I), 9322 data] and wR(F_2) = 0.1182 (all data); GOOF =1.060.

S53: Crystal data for compound **18**

$C_{21}H_{15}F_2N_5O$, M = 391.38, T = 100.0(10) K, λ = 0.71073 Å, trigonal, space group R3cH, a = 25.1585(8) Å, c = 15.4454(6) Å, V = 8466.4(6) Å^3, Z = 18, D_c = 1.382 Mg M^3 μ = 0.103 mm^{-1}, F(000) = 3636, crystal size 0.42 × 0.29 × 0.16 mm; θ_{max} = 33.41°, 23922 reflections measured, 6267 independent reflections (R_{int} = 0.0419); final R = 0.0431 [I > 2σ(I), 4689 data] and wR(F_2) = 0.1003 (all data); GOOF =1.043.

S54: ORTEP drawing of compounds **5** and **6**

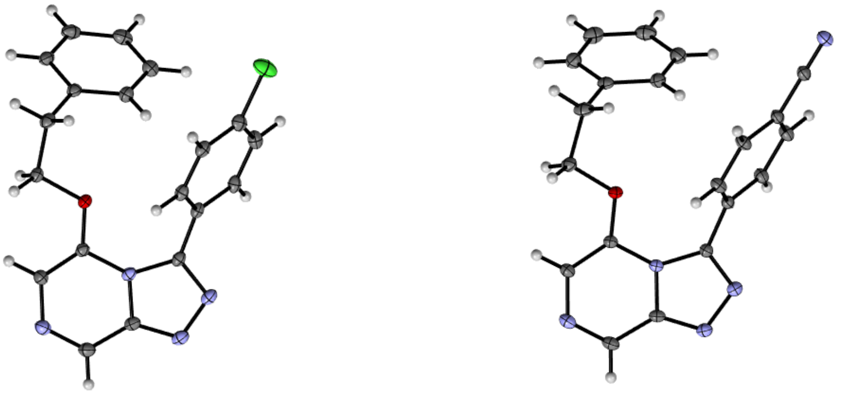

References

1. Sheldrick, G. M. *Acta crystallogr., C Struct. chem.* **2015**, *71*, 3-8.
2. Macrae, C. F.; Bruno, I. J.; Chisholm, J. A.; Edgington, P. R.; McCabe, P.; Pidcock, E.; Rodriguez-Monge, L.; Taylor, R.; van de Streek, J.; Wood, P. A. *J. Appl. Crystallogr.* **2008**, *41*, 466-470.
3. Farrugia, L. *J. Appl. Crystallogr.* **1999**, *32*, 837-838.
4. Duffy, S.; Avery, V. M. *Am. J. Trop. Med. Hyg.* **2012**, *86*, 84-92.
5. Fletcher, S.; Avery, V. M. *Malaria Journal* **2014**, *13*, 343.
6. Johnson, D. J. G.; Jenkins, I. D.; Huxley, C.; Coster, M. J.; Lum, K. Y.; White, J. M.; Avery, V. M.; Davis, R. A. *Molecules* **2021**, *26*, 2421.
